# Multifunctional Delivery Systems for Peptide Nucleic Acids

**DOI:** 10.3390/ph14010014

**Published:** 2020-12-25

**Authors:** Stefano Volpi, Umberto Cancelli, Martina Neri, Roberto Corradini

**Affiliations:** Department of Chemistry, Life Sciences and Environmental Sustainability, University of Parma, 43124 Parma, Italy; stefano.volpi@unipr.it (S.V.); umberto.cancelli@unipr.it (U.C.); martina.neri1@studenti.unipr.it (M.N.)

**Keywords:** peptide nucleic acids, delivery, nanoparticles, conjugates, multifunctional systems

## Abstract

The number of applications of peptide nucleic acids (PNAs)—oligonucleotide analogs with a polyamide backbone—is continuously increasing in both in vitro and cellular systems and, parallel to this, delivery systems able to bring PNAs to their targets have been developed. This review is intended to give to the readers an overview on the available carriers for these oligonucleotide mimics, with a particular emphasis on newly developed multi-component- and multifunctional vehicles which boosted PNA research in recent years. The following approaches will be discussed: (a) conjugation with carrier molecules and peptides; (b) liposome formulations; (c) polymer nanoparticles; (d) inorganic porous nanoparticles; (e) carbon based nanocarriers; and (f) self-assembled and supramolecular systems. New therapeutic strategies enabled by the combination of PNA and proper delivery systems are discussed.

## 1. Introduction

### 1.1. Peptide Nucleic Acids and Their Uses

**Figure 1:**
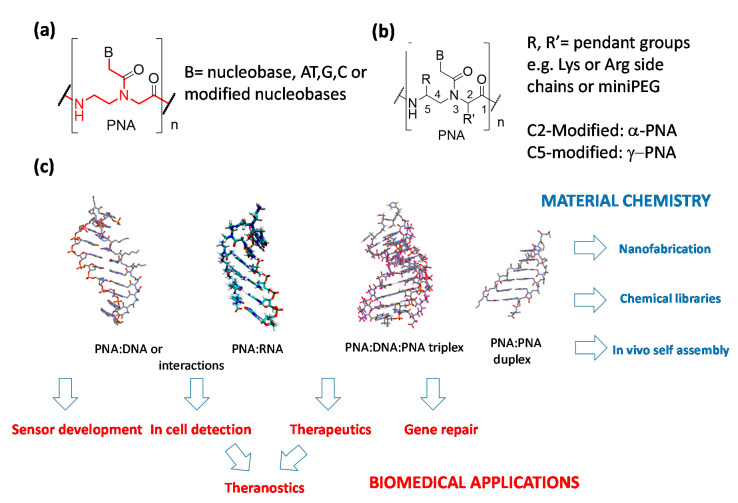
Schematic structures of (**a**) PNA and (**b**) modified PNAs with pendant side chains, and (**c**) types of complexes formed with DNA, RNA, and PNA and general applications derived from these interactions. The structures reported in (**c**) are 3D models derived from Protein Data Bank (entries inr8, 176d, 1pnn, and 1pup, respectively).

Peptide nucleic acids (PNAs, Figure 1) are DNA analogues in which the sugar-phosphate units connecting the nucleobases are replaced by *N*-(2-aminoethyl)glycine moieties [1]. These molecules are excellent binding partners for cognate DNA and RNA strands, being able to exploit both the canonical Watson–Crick (WC) base pairing to form stable duplexes [1,2] and a combination of WC and Hoogsteen hydrogen bonding to be arranged with their counterpart in triple-helical adducts [3], eventually performing dsDNA strand invasion [4,5]. PNAs and their open-chain analogs (bearing modifications in the polyamide backbones as indicated in Figure 1b) have been used in a series of important applications [6] encompassing drug development [7,8], RNA and DNA detection for diagnostics [9,10,11], nanofabrication [12,13], and chemical encoded libraries [14] (Figure 1c). PNAs are also particularly suited for the development of sensory systems (Figure 1c), thanks to their high stability in biological media [15] and to their high fidelity in the recognition of relevant DNA and RNA sequences [16]. In fact, they are able to tightly bind complementary nucleic acids with high selectivity, presenting a particular ability to discriminate mismatched sequences even in the presence of a single mismatched base (i.e., in point mutations or single nucleotide polymorphisms). In this review, however, we will focus only on the applications of diagnostics in which PNA probes are delivered into cells (in vitro or in vivo detection), leaving to the readers some general review for their use in the sensory sector [17,18]. Another application of PNAs which has recently attracted increasing attention is their use as “smart” materials for self-assembly, nanofabrication, and computing, as they are able to form PNA:PNA duplexes which can be easily modulated in stability and helical handedness [18,19,20].

**Figure 2:**
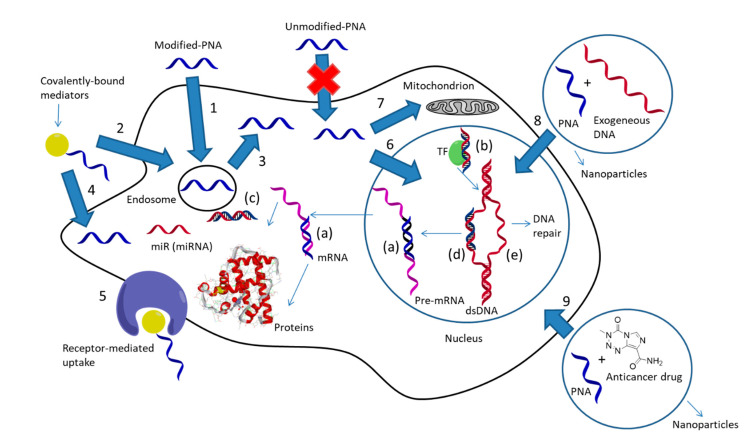
Uses and cellular delivery pathways for PNAs and PNA-containing systems described in this review; letters refer to PNA uses and numbers (arrows) refer to delivery pathways. Function: (**a**) antisense block of splicing or translation by targeting mRNA; (**b**) decoy strategy for scavenging transcription factors by PNA-DNA chimera; (**c**) anti-miR approach to block short non coding RNA (microRNA or miR), since the effect of miR is to down-regulate the mRNA translation, blocking miR has the effect of enhance the protein production (unlike antisense and anti-gene approaches); (**d**) anti-gene approach for blocking transcription by targeting dsDNA; (**e**) genome editing using PNA and exogeneous DNA. Delivery pathways: 1-direct cell permeation; 2-carrier mediated cell uptake with endosomal pathway; 3-endosomal escape; 4-carrier mediated permeation with cytoplasmic localization; 5-receptor mediated uptake; 6-nuclear delivery; 7-delivery to the mitochondria, thus targeting mitochondrial DNA (mtDNA); 8-nanoparticle mediated co-delivery of PNA and exogenous DNA; 9-nanoparticle mediated co-delivery of PNAs and drugs.

Being very efficient tools for modulating gene expression both in vitro and in vivo, PNAs and their analogues have been proposed as antisense molecules capable of blocking mRNA or correcting aberrant pre-mRNA splicing (Figure 2a) [21,22,23], a field that has recently boosted research on novel antimicrobial agents [24,25,26], as anti-gene agents, thus blocking transcription from DNA to mRNA (Figure 2b) [27,28,29], as anti-miR agents, blocking this important regulative class of non-coding RNAs (Figure 2c) [30,31,32,33], and as “decoy” molecules capable of sequestering transcription factors (Figure 2d) [34]. It should be noted that the antisense action of PNAs occur by steric blockage of the corresponding mRNA [35,36,37], without the occurrence of enzymatic digestion as instead observed in the RNAse-H- or RISC-mediated cleavage of other antisense oligonucleotides and siRNA. Similarly, PNA-based anti-gene or anti-miR systems rely only on their tight binding to their targets, and hence both their binding ability and intracellular concentration should be maximized to obtain a therapeutic effect. Another important and relatively recent use of PNAs—especially those able to form triplexes with target DNAs (i.e., bis-PNAs and tail-clamp PNAs)—is the induction of homologous recombination processes with an exogenous DNA segment, leading to genome editing or “repair” (Figure 2e) [38].

One of the most important issues in the development of PNA technology for biological applications, either for therapy or in vivo diagnosis, is their poor uptake by target cells. In order to solve this drawback, the various approaches that have been considered (Figure 2) can be differentiated into: (a) cellular delivery, (b) specific delivery in the subcellular compartment, and (c) tissue delivery for in vivo studies. In this review, we will focus on the use of multifunctional and multicomponent delivery systems to enable the cellular uptake of PNAs, with the aim to give a general view on this subject that update what reported in previous reviews [39] or extend more recent works describing only some aspect of nanoparticle-based PNA transport [40,41]. Additionally, the description of the approaches exploiting these types of oligonucleotides as building blocks for multifunctional materials will be discussed, with a particular emphasis on recent works and concepts unveiling the enormous possibilities opened by the rational use of PNAs as components for “biomolecular engineering” [42].

### 1.2. Approaches for Cellular PNA Delivery and PNA-Mediated Delivery

The strategies for PNA delivery described so far were based on several distinct approaches: (a) direct delivery in the cell culture or by microinjection; (b) conjugation with carrier molecules, mainly, but not solely, peptides; (c) delivery with liposomes and lipophilic particles; (d) incorporation into polymer nanoparticles; (e) interaction with non-polymeric organic carriers; (f) incorporation into inorganic or carbon-based nanostructured materials. Combinations of these approaches (e.g., using inorganic nanoparticles and peptide carriers) have also been described, giving rise to multifunctional systems.

The use of PNAs as simple additives in cell cultures is generally not a productive approach, though it worth mentioning some exceptions: in fact it has been reported that neurons are more permeable to PNAs than other cell types [43] and this leads to possible non-zero activity of control experiments made with naked PNAs when the effect of carrier-mediated delivery is studied. Internalization into eukaryotic cells is in general easier than in prokaryotes, the latter being dependent on the type of the targeted bacteria and the composition of their cell-wall (Gram-positive or Gram-negative); in particular, it has been shown that the lipopolysaccharide layer in Gram-negative bacteria acts as a barrier for PNA delivery [44] and recently some approaches have been described to overcome this problem, thus paving the way for fully-effective antimicrobial PNAs [20,21,22].

In the earlier reports, unmodified PNAs were introduced into cells through mechanical or electrical techniques such as electroporation [45,46], microinjection [36], co-transfection with complementary DNAs [47], or direct permeabilization of the cell membrane [48]. These approaches were useful to demonstrate that PNAs can in principle be delivered into cells and modulate the expression of target genes, but, being highly invasive, they were not suitable for large-scale or in vivo experiments.

The modification of PNA derivatives has been explored, in order to rely on milder conditions for achieving their direct internalization in target cells. The functionalization of the pseudo peptide backbone on the C(2)- or C(5)-position with positively charged alkylammonium- [49,50] and alkylguanidinium units [51,52,53,54] or mini-PEG chains [55] gave good results, yielding molecular entities which were effectively taken up by different cell lines. The use of modifications on the backbone can also afford additional advantages in terms of affinity and selectivity for cognate DNA or RNA strands [56,57,58], thus potentially combining an improved intracellular transport with a higher biological activity, but, on the other hand, it requires longer and challenging procedures for the synthesis of the corresponding PNAs. 

A further point that has only recently been addressed is the possible (inverse) use of PNAs as carriers. In fact, although PNAs are not internalized in most cells, they can be easily conjugated with specific peptides and ligands able to promote their uptake; thus the tight binding of a peptide-PNA conjugate to a DNA or RNA tract can be used as a simple way to introduce a complementary nucleic acid without the need of covalent modifications on the carried moiety [59,60]. The labelling of nanoparticles for imaging through PNAs be performed in this way, yielding multicomponent and multifunctional systems [61]. Thus, in the field of multifunctional delivery systems it can be foreseen that PNA-mediated conjugation will also play a very important role in the creation of “smart nanosystems”.

### 1.3. PNA Localization

The intracellular localization of PNAs, once that cellular uptake has been achieved, is another critical point to be addressed. In fact, in many examples, the action of the internalized PNAs is limited by the endosomal entrapment, which leads to the need of higher effective concentrations than those required by direct cytosolic delivery for a significant activity. Strategies for endosomal escape (e.g., by calcium ions or chloroquine treatment) [62], when used, are thus able to substantially increase the activity of PNAs as antisense or anti-miR agents. Micromolar concentrations are necessary to observe a biological effect for PNAs that undergo entrapment into endosomes, whereas direct delivery to the cytosol or endosomal escape can lower this value in the sub-micromolar range [63]. 

Delivery to the nucleus is also desirable when an anti-gene strategy is used; this can be achieved through conjugation with nuclear localization signals peptides or small drugs. Nuclear localization signal (NLS) is a peptide enabling a cargo to be transported into the nucleus through binding to importin α, although it is not a good carrier for crossing external cell membranes. Multifunctional vehicles bearing a cellular delivery systems and a NLS-peptide have been developed to solve this problem [64]. More specific targeting of internal cellular compartments can be achieved using specific carriers. For example, PNAs were shown to be delivered to mitochondria through conjugation with a triphenylphosphonium moiety, thus exerting inhibitory effects on mitochondrial DNA and disrupting mitochondrial fusion [65], and even better results were obtained by using a COX VIII pre-sequence [66]. 

The pharmacokinetics of PNAs in vivo have been evaluated in some studies after intra-venous (IV) administration, pointing out a PNA distribution half-life of 3 min and a clearance half-life of 17 min [67]. One difficult task is the permeation of the blood-brain barrier (BBB) for treatment of brain tumors and diseases, though some specific delivery systems able to cross the BBB were reported [68].

For all these reasons, it is clear that the possible success of the most promising applications of PNA-based technologies strongly depends on the effectiveness of their delivery to the target organs or cell-type, both in terms of internalization and of localization in the proper cellular compartment (e.g., cytosol, nucleus, mitochondria, etc.). The different carrier types will, therefore, be discussed in terms of their performances in these tasks, starting from the classic PNA delivery systems, with some updates from recent literature, and moving to the most recent approaches exploiting multifunctional and multi-component vehicles (i.e., able to perform different task at the same time).

## 2. PNA Conjugates

### 2.1. Small Molecule Ligand Conjugation

The covalent conjugation with (macro)molecules able to traverse cell membranes has been widely explored over the years for PNA transport. Lipophilic adamantyl groups attached on the C-term of 15-mer PNAs were reported to facilitate their uptake in cervical, lung and breast cancer cells [69], despite the delivery was more effective in the presence of liposomes and cell-type dependent, while the conjugation of triphenylphosphonium cations in the same position was used for targeting the mithocondria of human fibroblast cells [70]. *N*-acetyl galactosamine (GalNAc) units can be instead used for targeting human hepatocytes, by exploiting the recognition of the highly-specific asialoglycoprotein receptor (ASGPR) expressed on their membrane. In fact, a sequence complementary to the microsomal triglyceride transfer protein (MTP) gene, once connected to a divalent ligand containing two GalNAc units on the N-term, was reported to promote the downregulation of its biological target in both human [71] and murine liver cells [72], as a results of a good internalization. Interestingly, a similar trivalent adduct was recently found to effectively penetrate into HepG2 cells, but the delivery of the PNA oligomer was improved when the GalNAc groups were placed on three consecutive C(5)-modified PNA monomers instead of a branched spacer at the N-term, thus resulting in a linear orientation of the glycosyl moieties [73]. A structure-dependent activity was also reported for a system based on modified 2, 4-diaminobutanoic acid (Dab) dendrons, conjugated to a PNA to be delivered in HeLa cells [74]. A luciferase assay was used to monitor the internalization of the PNA-dendron adducts, reporting a higher performance when guanidinium moieties were used as terminal groups of the dentritic structure in place of ammonium ones, with a further improvement of the uptake upon increase of both the degree of functionalization and the addition of lipophilic units (i.e., longer alkyl spacers, fatty acids or aromatic substituents), although at the expense of a higher toxicity. Vitamin B12 was instead used as a tool for targeting Gram-negative bacteria [75], leading to the inhibition of the *E. coli* growth when coupled with an antisense PNA [76]. Also in this case, the transport of the tested PNAs was affected by the structure of the linker used to connect vitamin B12 to the N-term of the sequence and to the geometry in which the two molecules were connected, with the best results reported when the PNA oligomer was anchored on the 5′-OH of the vitamin [77]. 

Conjugation with phospholipid units [78] has also been shown to improve PNA delivery, though with endosomal pathway, as demonstrated by the improvement of the biological effect in the presence of chloroquine.

### 2.2. Peptide Conjugation

One of the most widely used approaches for cellular delivery of PNA involves the conjugation to cell-penetrating peptides (CPP) also known as protein translocation domains (PTD) or Trojan peptides (TP). CPPs can be generally classified into two categories: cationic CPPs, usually rich in positive-charge amino acids such as lysine or arginine and amphipathic peptides, whose structure allows a direct translocation across the plasma membrane [79,80]. The comprehensive description of the delivery of PNA-CPP adducts is out of the scope of this review, so only the most relevant results obtained in this field will be listed in the following lines, since PNA-CPP conjugates can also be components of multifunctional systems. Naturally-occurring CPPs, such as penetratin and transportan, in an early study, were reported to improve the ability of an antisense PNA sequence for limiting the expression of galanin receptors in rats [81]. In other investigations, penetratin was combined with a triplex-forming PNA to promote the gene editing of hematopoietic stem cells (HSC) in mice, with a retention of the targeted modification for at least two weeks after systemic administration [82]. In contrast to the typical design in which the given CPPs are bound to one extremity of a PNA oligomer, Tan et al. reported a structure in which the transactivation of transcription peptide from human HIV-1 virus (TAT) was flanked by short C(5)-modified PNA segments having complementary sequence [83]. In this way, the TAT peptide was forced to form the loop of a chimeric hairpin, whose uptake in A549 cells was 10-folds higher than that of an analogous construct not able of installing intramolecular base pairing. Moreover, the addition of an overhang on the PNA-TAT hybrid allowed for the conjugation of supramolecular cargos linked to a complementary DNA strand, facilitating their delivery in target cells. 

The pH-low insertion peptide (pHLIP)—a molecule able to transport different cargoes by directly crossing the cell membrane in cancer acidic environment [84], thus bypassing endocytosis—was bound to a PNA targeting the oncogenic microRNA-155 (miR-155) in a mouse model, leading to a reduced progression of lymphoma thanks to the correct accumulation in this type of solid tumor [31]. 

Various examples of artificial CPPs have been designed for PNA delivery as well, finding applications as gene modulators [37,85,86] and as antibacterial agents [87,88]. Among them, polyarginine and polylysine pendants are probably the most commonly used modifications for these purposes. For example, octa-arginine PNAs showed good activity against different type of microRNAs (miRs) in proper cell cultures, such as miR-145 and miR-221. In the former case, the inhibition of the biological target led to an increased expression of the cystic fibrosis conductance regulator (CTFR) in Calu-3 cells [89], while in the latter example the sequestration of the given miR promoted a higher expression of the p27Kip1 gene in the breast cancer MDA-MB-231 line [90]. Both octa-arginine and tetralysine tails were instead found to induce a PNA-mediated decrease of hepatitis B virus replication after intravenous administration in ducklings, with better results in terms of sequence selectivity when the polylysine moiety was conjugated to the given antisense oligomer [91,92].

An interesting approach for the rational design of CPPs with improved internalization activity was presented in a recent paper by Kauffman et al., by taking as reference the sequences of penetratin and TAT peptides [93]. The authors designed a library of 8192 derivatives with mixed sequences by a synthetic molecular evolution (SME) technique, and the internalization of the corresponding PNA-CPP adducts in HeLa cells was monitored tanks to a dedicated luciferase assay. This screening pointed out that most of the daughter peptides induced a significant increase of cell uptake after a single iteration of SME, with an increase of internalization of two order of magnitude with respect to the parent sequences in the best case.

Despite PNA-CPPs conjugate have the potential to become an effective tool for biomedical applications, some drawbacks have still to be overcome for their extensive use. First of all, excluding some isolated case [31,94], their cellular uptake is suggested to occurs mainly by endocytosis [95], with a limited escape from endosomal and lysosomal vesicles due to the high cationic content of their peptide moiety [96,97]. Additionally, the conjugation of PNAs to CPPs could produce significant levels of toxicity, which hamper the development of protocols for in vivo applications [98]. Finally, although convenient strategies of conjugation have been refined for both solution and solid phase synthesis, the production of PNA-CPP hybrids remains relatively labor-intensive, limiting their use at a large scale.

For these reasons, alternative technologies have been explored for PNA delivery, consisting in the non-covalent association of these oligonucleotides with nanoscaled vehicles and nanoparticles [40,41]. The use of nanoparticles and related systems have some advantages with respect to the covalent association with small- and macromolecules, including versatility of the loading procedures, increased rates of internalization and release, and ease of modifications for obtaining multifunctional systems. Accordingly, the following section will be focused in a detailed description of the nanoparticle-based techniques for improving the permeability of PNA toward cell membranes. 

## 3. Lipid and Liposome-Based Approaches

One alternative to conjugation is the incorporation of PNA into lipid-based transfection systems. One strategy developed by Corey and co-workers and used in many applications for PNAs was the use of cationic transfecting agents such as lipofectamine, polyethyleneimine (PEI), or other cationic transfecting agents. Since neutral PNAs do not spontaneously interact with these systems, partial complexation with a complementary DNA was used in order to load them, and an efficient transfection of antisense PNAs was achieved in this way [99]. An alternative is represented by conjugation of PNA with anionic peptides, such as oligo-aspartate, with facilitate the incorporation into lipofection carriers [100]. 

Liposomes and lipid nanoparticles are widely used for the delivery of insoluble drugs, but many liposomal systems for the transport of nucleic acids into cell have been also developed; some of these are very effective and have been the basis for the success of FDA approval of antisense drugs, and, more recently, of the first siRNA treatment for the dowregulation of gene expression in tumors [101,102]. Liposome nanovesicles can be finely tuned to be pH sensitive or stealth, the latter being particularly suited for developing drug delivery systems able to contrast the detection and degradation of the carried cargo by the phagocyte cells of the reticuloendothelial system [103].

The use of liposomes for PNA delivery has been described by several authors: the lipid-mediated carriage into cells was performed by conjugation of PNA derivatives with apolar moieties (such as the adamantly unit mentioned above [69]) or with modified monomers based on lysine, leading to a cellular uptake that was however dependent on both the PNA structure and the targeted cell type [61]. The encapsulation of unmodified anti-miR PNAs inside liposomal nanosystems was instead achieved and effectively used for their delivery inside K562 tumor cells [104], and a significant anti-miR effect was observed in the range 0.5-4 mM PNA concentration. Subsequently, the liposome composition was optimized by using DOPC/DOPG, cholesterol, and DSPE-PEG 2000 [105]. 

Another approach to improve cell penetration of oligonucleotides consists in functionalizing the surface of PEGylated liposomes with cell penetrating peptides [106]. Alternatively, a multifunctional delivery system based on Phospholipase A-sensitive vesicles was recently described for octa-arginine PNAs [107]. In this system, the enzyme secreted by tumor cells was used to disrupt the phospholipid structure and to release the cargo. However, the liberated PNA could be hindered by the fusion of the PEG layer with plasma proteins (or serum proteins), which can form a protein corona structure on the liposome surface. Another problem of liposomes is constituted by the aggregation of the particles and their non-uniformity in terms of size and lamellarity and, recently, alternative lipid carriers have been developed, such as quatsomes, composed of cationic lipid/cholesterol mixtures [108], or niosomes, composed of non-ionic surfactants [109]. Another particular class of vehicles, used in combination with PNA, is represented by bidimensional (discoidal) nanostructure, called by the authors “bicelles”; these structures were shown to induce a 5–10-fold higher cellular uptake than spherical vesicles. Anti-miR-210- and anti-miR-155 PNA-loaded bicelles were effectively delivered into HeLa cells, inducing anti-miR effects. The mechanism of internalization was found to follow different pathways, and for this reason it was proposed to be more effective [110].

## 4. Polymer Nanoparticles and Carriers

### 4.1. Polylactic Co-Glycolic Acids (PLGA)

**Figure 3:**
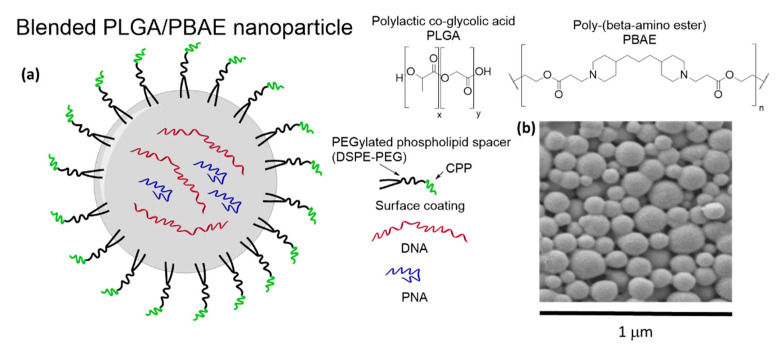
(**a**) Schematic representation and (**b**) SEM image of blended PLGA/PBAED nanoparticles coated on their surface with a CPP and loaded with generic DNA and PNA cargoes. Panel (**b**) is adapted with permission from [113]: Fields et al. *©* 2014 WILEY-VCH Verlag GmbH and Co. KGaA, Weinheim.

PLGA is a copolymer composed by a mixture of lactic- and glycolic acid esters (Figure 3a). Being non-toxic and fully-biodegradable, it is approved by both the US Food and Drug Administration (USFDA) and the European Medicines Agency (EMA) and it is widely used for the delivery of drugs [111]. PLGA slowly is degraded by hydrolysis of its polyester framework under physiological conditions, allowing for the controlled release of previously-loaded molecular cargoes [112].

The rate of degradation and other physical features can be tuned as a function of the ratio between the lactic and glycolic building blocks composing the polymer or by post-synthetic functionalization of its surfaces with multifunctional synthons, such as polyethylene glycol (PEG), CPPs, folate, and other molecules [114,115]. Additionally, the properties of this material can be further customized by co-formulation with other polymeric compounds such as poly-(beta-amino ester) (PBAE) [116], which provide the possibility to create pH-responsive composites being stable under neutral conditions and readily degradable at pH < 6.5 [117]. This property can be used to release a payload in the acidic environment of tumors or endosomes from blended nanoparticles [117,118,119,120], and to promote the disruption of endosomal vesicles. Although positively-charged components can increase the toxicity of the final material, the use of 15% of PBAE in mixture with PLGA is typically suitable for biological applications [120]. Both regular- and blended PLGA nanoparticles (PLGA NPs) have been successfully used for the delivery of proteins, nucleic acids, and other (bio)molecules, often inducing a significant increase of their activity as a result of a higher cell internalization and improved pharmacokinetics [111]. 

PNAs can be loaded in PLGA NPs by double emulsion solvent evaporation protocols in higher yields with respect to most of the nucleic acid families [121], and the encapsulation of oligomers against miR-155—a microRNA overexpressed especially in cancers of hematopoietic origin—was reported in two different studies. In the first [121], PLGA NPs were coated with an arginine-rich CPP via a PEGylated phospholipid linker (DSPE-PEG) [115] to increase the association with target cells. A decrease in the bioavailability of miR-155 by 40% was observed, and the release of the nucleic acid cargoes into cells was proposed to occur by both diffusion and hydrolysis of the PLGA architecture. Naked NPs were also tested in a control experiment in which a lower inhibition of the target miR was achieved, thus confirming the role of the peptides coating the surface in improving the targeting of KB cells. In a second report the same authors found that analogous systems could be effectively used for targeting miR-155 in vivo on a NesCre8 mir-155LSLtTA murine model [122]. The NPs were decorated with penetratin to increase cell-penetration and administered twice through the tail vein; this treatment was able to inhibit the growth of the targeted tumors by 50%. The nanostructured vehicles were accumulated preferentially around the lymphomas with respect to other tissues thanks to the “enhanced permeability and retention effect” (EPR)—a phenomenon which promotes the passive targeting of tumors due to the defective architecture of their blood vessels [123]—and were retained on the spot for at least two days. A dose corresponding to 1.5 mg/kg of PNA loaded into NPs was sufficient to report a relevant therapeutic response, which was significantly lower than the doses required by alternative anti-miR treatments known at the time of the publication [124,125]. Another investigation was instead focused on the sequestration of miR-210 [126], overexpressed in several cancer cells in response to hypoxia, and associated to almost all solid tumors [127,128,129]. In this case, the activity of an unmodified derivative was compared with that of a C(5)-modified PNA (MPγPNA), bearing short polyethylene glycol chains (miniPEG) pendants on their backbone. MPγPNAs are known to exert an higher binding affinity for cognate DNAs or RNAs due to the insertion of chiral centers with appropriate stereochemistry, which conveniently pre-organize the pseudo-peptide backbone [130,131], possibly improving their features as antisense agents. In fact, mice engrafted with human HeLa cells experienced a significant delay in tumor growth after treatment with PLGA NPs loaded with the anti-miR MPγPNA, while the delivery of the unmodified analog behaved approximately as the control experiments, in which blank vehicles were used. Moreover, the intra-tumor administration of 0.8 mg/kg miniPEG-PNA was high enough to report an apparent anticancer effect without coating the surface of the nanoparticles with any CPP, thus improving the results reported above for the inhibition of miR-155 [122]. Similarly, the PLGA-based delivery of PNAs was also exploited for other antisense applications, targeted against the human CCR5 gene from the template strand of the genomic DNA [132], which is crucial for HIV-1 virus infection [133]. MPγPNA and a hybrid derivative functionalized at the C(5)-position with both mini-PEG pendants and butylguanidinium units, called MPγGPNA, were found to be more effective than unmodified PNAs for this purpose. In fact, the C(5)-modified oligomers, having a higher affinity for their target whit respect to regular PNAs [54,130,131], exhibited a higher activity due to a more efficient inhibition of the translational processes.

**Figure 4:**
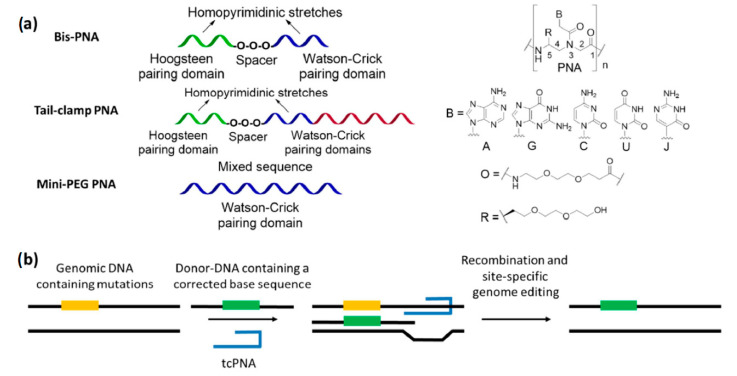
(**a**) Cartoon representation of the types of PNAs which have been used to perform site-specific gene editing with the help of PLGA NPs as delivery system; (**b**) schematic description tcPNA-mediated gene correction processes in the presence of a donor-DNA presenting the corrected base sequence.

The promotion of gene-editing processes (Figure 4) by triplex-forming PNAs is another field in which the PLGA NPs has been tested as delivery vehicles. 

Triplex-forming PNAs are oligomers able to invade the structure of double-stranded DNAs on their homopurine stretches, by installing contemporary Watson–Crick and Hoogsteen base pairs (Figure 4b). The formation of triple-helical adducts imparts a distortion in the target duplex which activate the DNA repair machinery, triggering the modification of defective genes in the presence of a 50–60 mer DNA fragment having the corrected sequence (donor-DNA) [38,134]. Among the various PNA derivatives, bisPNAs and tail-clamp PNAs (tcPNAs) have been used to induce gene-editing by triplex formation. The former are composed by two segments—covalently connected by a spacer—which are able to generate the required PNA/DNA/PNA triple helix with the target genomic DNA, while the latter can be considered as refined oligomers in which an overhang (the tail) is added to form an extended Watson–Crick pairing domain. The formation of a very stable complex with the genomic duplex is crucial to initiate the DNA modification procedures, since it prevents the displacement of the given PNAs by helicase enzymes [38]. In fact, tcPNAs express an increased gene-editing activity with respect to analogous bisPNAs [38,40,135], thanks to the presence of a larger Watson–Crick binding part which confer higher affinity and selectivity for a cognate duplex DNA [136,137]. However, a limitation of triplex-forming PNAs is the need of homopurine sites to correctly bind their target. This problem could be overcome by using C(5)-modified oligomers, which give rise to adducts with a sufficient stability to invade their double stranded counterpart without forming triple-helical adducts [138,139]. In particular, single-stranded PNAs bearing mini-PEG chains on their backbone have been reported to effectively promote gene-editing processes without sequence restrictions [140].

Correction of the IVS2-1 (G→A) mutation in the human β-blobin gene—a genome mutation causing β-thalassemia—was originally obtained in vitro by co-transfecting a donor-DNA and some bisPNAs with a nucleofection/electroporation system [141]. Subsequently, the correction of the same genomic site was performed by delivering the best bisPNA/donor-DNA couple into CD34+ human progenitor cells with PLGA nanoparticles [142]. Further investigations also indicated that the PLGA-based delivery system was superior to the previously reported nucleofection strategy [141], reporting a 63-fold increase of the gene-correction rate for the former internalization method. Moreover, the retention of the genome modification was reported in both erythroid- and neutrophil-differentiated cells after 30 days of culture of NPs-treated progenitors. The possibility of correction for the same mutation was also evaluated in vivo in humanized mice, with a retention of the edited gene both 5 and 10 days post-treatment in blood cells taken from bone marrow, spleen and lungs [143]. The gene-editing procedure was further validated by using a green fluorescent protein (GFP) fusion gene bearing the human β-globin IVS2-654 (C→T) mutation, whose correction was promoted by NPs containing an appropriate tcPNA/donor-DNA couple to restore the expression of the fluorescent protein in transgenic mice [143]. This procedure was then improved using C(5)-miniPEG PNAs as tools to induce gene correction [140], and tested in vivo [144] for the in utero targeting of the IVS2-654 (C→T) mutation in β-thalassemic murine fetuses. Alleviation of anemia with respect to the control animals was induced, with an increase of in-utero survival from 69% to 100%. In bone marrow, the gene-editing frequency was around 9% three days after the administration of the NPs and 6% 15 weeks later, indicating an effective retention of the modification in adult mice. The off-target effects were extremely limited, especially in comparison with other enzymatic technologies like CRISPR/CAS9 [145]. 

Further studies demonstrated the feasibility of this approach for correcting: (a) the CCR5-Δ32 mutation, involved in HIV-resistance [133,146] in different in vitro and in vivo investigations [143,147], over-performing the selectivity of other nuclease-based agents known at the time [148]; (b) the defective cystic fibrosis transmembrane conductance regulator (CFTR) [149], using vehicles coated with a synthetic CPP called MPG [120] on a model system bearing defective a EGFP fusion gene [113], and subsequently for the correction of the real CFTR gene both in vitro and in CF mice [150]. In the latter case, the delivery of a tcPNA/donor-DNA combination by naked PLGA NPs in human CFBE cells yielded a modification of the target F508del mutation in the 0.5–0.96% range, while the use of MPG-decorated PLGA/PBAE vehicles allowed for an improved gene-editing performance and to restore a normal chloride efflux in 25% of the tested cells. The in vivo correction of the murine F508del was also demonstrated after intranasal administration of the decorated NPs loaded with the same oligonucleotides.

### 4.2. Cationic Shell-Cross-Linked Knedel-Like (cSCK) Nanoparticles

**Figure 5:**
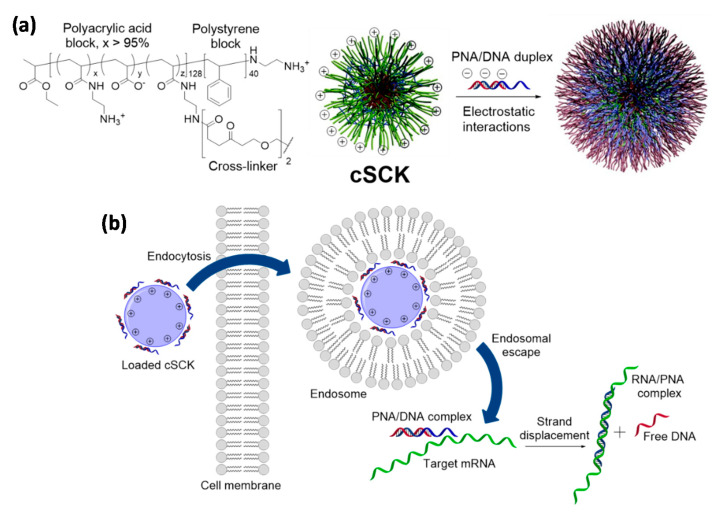
(**a**) Loading of cSCK nanoparticles with PNA/DNA complexes by exploiting the electrostatic interactions installed between the surface of the positively charged nanoparticles and the negatively charged adducts; (**b**) suggested mechanism of internalization of PNA/DNA-loaded cSCK and release of the nucleic acid cargoes into the cytoplasm, followed by the formation of a complex including the PNA component and a generic mRNA target thanks to the displacements of the DNA strand from the original duplex. Panel (**a**) is adapted with permission from [153]: Fang et al. *©* 2009 American Chemical Society.

SCKs (Figure 5) are a large family of cross-linked block copolymer micelles which are obtained by a combination of radical and ring-opening polymerizations, chemical modifications and self-assembly processes [151]. Their use in biotechnology and medicine have been mainly focused on the intracellular delivery of relevant payloads, such as small drugs, diagnostic agents, and nucleic acids. The physicochemical properties of these nanoparticles depend on the copolymers selected as building blocks and the chemical compounds used for their cross-linking and surface decoration, giving the possibility to create tailored carriers able to host different type of molecules within their core-shell architecture [151,152]. 

Regarding PNAs, despite the conjugation with regular cross-linked nanoparticles has been explored for nanotechnology applications [154], the reports regarding the improvement of their cellular uptake have relied on cationic SCKs (cSCKs). cSCKs can be obtained from a copolymer containing adjacent polystyrene and polyacrylic acid segments (the core and the shell, respectively) by functionalization of the carboxylic units belonging to the latter block with *N*-Boc-ethylenediamine, followed by removal of the protecting groups, micellization and crosslinking of the structure [155]. In this way, it was possible to reverse the original surface charge of the nanoparticles and promote the association with a negatively-charged plasmid DNA, which was carried into HeLa cells with a higher efficiency than that of a commercially available transfection system [155]. This report was then taken as reference for the delivery of PNAs, since it was reasoned that the amino pendants of cSCKs could provide an anchoring point for their covalent conjugation and that PNA/DNA duplexes could still be adsorbed by electrostatic interaction with the surfaces of these nanoparticles. A PNA able to target an aberrant splice site on the luciferase gene pLuc705 [156], in fact, was transfected in HeLa by cSCKs either in combination with a shorter DNA strand or via the attachment on the amine units of the nanoparticles with a cleavable disulfide spacer [153]. In the first case the PNA cargo was released from the carrier by strand displacement once put in contact with the target gene, while in the latter the reducing environment found in the cytoplasm after transfection promoted the liberation of the PNA by breaking the disulfide moiety. For the PNA/DNA complex, luciferase and PCR assays reported that cSCKs were superior to the commercially available agents Lipofectamine 2000 and Polyfect since their delivery ability was associated with a lower toxicity for cells, thus allowing the treatment with higher amounts of both the carrier and the nucleic acid cargoes. The PNA-ligated nanoparticles performed similarly to those loaded with the PNA/DNA duplex under analogous experimental conditions, but transported the PNA payload much better than a classical polyarginine-based CPP or cSCKs functionalized with a non-cleavable linker. Mechanistic investigations indicated that the nanoparticles were able to promote endosome destabilization by sponge effect [157], remaining, however, entrapped in these compartments due to their relatively large dimension. Hence, it was proposed that the disulfide-modified PNA was selectively released by exposure to the reducing agents of the cytoplasm, tacking advantage from both an improved endocytosis and a more effective endosomal escape for a higher transfection efficiency. 

The cSCK-mediated delivery of PNAs was also used for the in vitro and in vivo diagnosis of acute lung injury (ALI), by selecting as target the mRNA transcript of inducible nitric oxide synthase (iNOS). ALI is a widespread inflammatory condition of the lung that is associated with acute respiratory distress syndrome [158]. iNOS is one of the overexpressed mediators in this pathology and it is responsible of the exceeding production of nitric oxide (NO) in activated lung macrophages, thus causing tissue damage [159,160]. iNOS mRNA-targeted nanoparticles were used for the indirect detection of NO in cells or tissues and to obtain an early diagnosis of the disease. For this purpose, different PNA-based probes were designed and then paired with a cognate DNA strand for a successful cSCKs-mediated delivery, creating fluorescence switches [161], strand displacement-activated probe [162], or a binary FRET probes [163] able to bind segments of the iNOS mRNA with a dissociation constant in the nM range [164]. An alternative strategy was explored by using the radiolabeled PNA called ^123^I-PNAYR9 [165]. This compound included an anti-iNOs mRNA sequence and an additional peptide chain containing a tyrosine residue for the functionalization with radioactive ^123^I and a nona-arginine segment for facilitating endosomal and lysosomal escape of untargeted probes. Additionally, in this case, the PNA was electrostatically adsorbed onto cSCKs after complexation with a cognate DNA and the incubation of activated RAW264.7 cells resulted in the effective uptake of the probe, which was significantly more retained than a mismatched analog delivered with the same hybrid vehicles. Similarly, mice treated with ^123^I-PNAYR9/DNA/cSCK composites accumulated the radiolabeled PNA in lungs once that the animals were induced for the overexpression of iNOS. The administration of ^123^I-PNAYR9 in the absence of nanoparticles resulted in a rapid clearance of the probe, evidencing the importance of cSCKs for an effective retention in the target tissue, while the use of carriers loaded with a mismatched PNA gave a significantly shorter maintenance of radioactivity thanks to the good sequence selectivity for the mRNA of interest. On the other hand, in the tested experimental conditions, the authors reported that the signal output generated by the different uptake of the full-matched and mismatched PNAs was not sufficient for diagnostic imaging.

### 4.3. Natural Occurring Biopolymers

In recent years, other naturally occurring materials have been used to attempt the intracellular delivery of PNAs, including chitosan, a copolymer composed by glucosamine and N-acetyl glucosamine that can be obtained by deacetylation of chitin [166]. Despite its high biocompatibility, normal chitosan is not suitable to work as drug carrier because of its poor solubility in water at physiological pH. However, the functionalization of its backbone with appropriate units can help to overcome this problem and to impart useful assembly properties for intracellular delivery [167]. In fact, micelles formed by amphiphilic chitosan derivatives have been used for the transport of different payloads, as reported in one study for PNAs [168]. In this investigation, a library of *N,N,N*-trimethylammonium-*O*-alkyl chitosan analogs (TMACs) were arranged in spherical nanoparticles and subsequently tested for carrying mixed-sequence PNAs into HeLa cells. The length of the apolar pendants on the oxygens of their backbone strongly affected the encapsulation, toxicity, and transfection properties of the chitosan-based micelles, with the achievement of the best results when a hexadecyl chain (cetyl) was used. In fact, the cellular uptake of the given PNAs in the presence of cetyl-TMACs was 66-fold higher than that obtained in the absence of nanoparticles, without the detection of significant hemolysis or cytotoxic effects.

## 5. Inorganic Nanocarriers

### 5.1. Zeolite Nanocrystals

Porous inorganic nanocarriers can be dispersed in water and have appropriate dimension to be used for the delivery and especially the co-delivery of different types of molecules, since their cavities are able to contain small drugs and they can be decorated on their surface with recognition elements for bio-macromolecules. For example, a widely used DNA-interacting dye (4′,6-diamidine-2′-phenylindole dihydrochloride, DAPI) and DNA oligonucleotides were delivered into HeLa cells thanks to modified zeolite-l nanocrystals, previously decorated with a biodegradable poly-l-Lysine (PLL) layer. Both the cargoes were then released upon degradation of the PLL layer [169]. Similarly, PNA derivatives were covalently attached to the surface of zeolite-L nanocrystals and co-delivered with small molecules such as DAPI to be released after PLL degradation. The additional inclusion of the insoluble dye *N*,*N*′-bis(2,6-dimethylphenyl)perylene-3,4,9,10-tetracarbodiimide (DXP), entrapped in the zeolites channels, allowed to follow the zeolite fate [170]. In this work, the role of the PLL layer, was also that of promoting cellular uptake; however, the use of the PLL coating could be avoided by conjugation of cationic PNAs to the zeolite-L surface. Furthermore, PNA:PNA duplexes could be used to assemble a dendrimer ‘stopcock’ at the surface, thus slowing the release process [171].

### 5.2. Mesoporous Silica Nanoparticles (MSNPs)

Mesoporous silica nanoparticles (MSNPs) represent a promising platform for various biomedical applications, such as bioimaging, biocatalysis and drug delivery. Their success is due to the good biocompatibility and degradability, in addition to their high loading capacity, the tunable pore structure, and the possibility to functionalize both the inner pore system and the outer particle surface. 

Since in 2001 the use of MSNPs as nanocarriers was first proposed to transport ibuprofen [172]—an anti-inflammatory drug—many efforts have been made to create multifunctional delivery platforms capable of encapsulating and carrying therapeutic molecules, such as peptides, proteins and nucleic acids. Moreover, the properties of MSNPs can be widely modulated by attaching molecular functionalities to their external and/or internal surfaces, including gatekeepers [173], targeting ligands [174], polymers [175], and tracking markers [176], with the aim to exploit their whole potential for nanomedicine developments. Together with an excellent biocompatibility [177], MSNPs possess a large surface area thanks to their internal pores, whose structure can be finely tuned by varying the parameters used in the nanoparticles synthesis [178]; their surface can be coated or modified in order to better fit payload entrapment, dispersion, and delivery [179]. Multiple co-delivery is also possible using MSNP, as demonstrated in the cases of siRNA and plasmid DNA [180].

**Figure 6:**
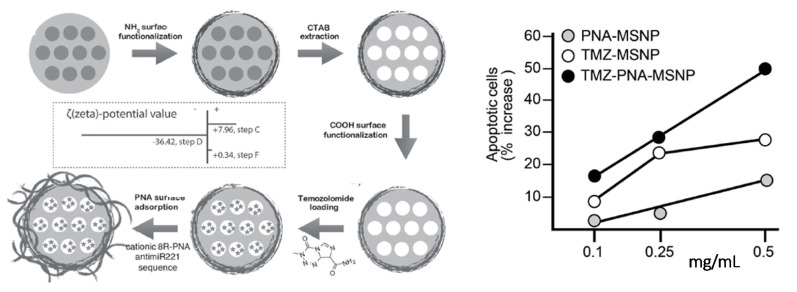
Functionalization of MSNPs and effect of MSNP-based treatments on cell apoptosis. Left panel: schematic procedure for the functionalization of PNA-TMZ-MSNPs. Right panel: induction of apoptosis after treatment of T98G cells with increasing amounts of TMZ-MSNPs, PNA-MSNPs, or PNA-TMZ-MSNPs, as indicated. Reprinted with permission from Bertucci et al. [181] *©* 2015 WILEY-VCH Verlag GmbH & Co. KGaA, Weinheim.

An anti-miR221 PNA conjugated with octa-arginine pendants was shown to be adsorbed onto MSNPs loaded with the chemotherapeutic agent temozolomide (Figure 6). The presence of the cationic PNA, in addition to improve the cellular uptake of the functional nanosystem, also served as gatekeeper to control the release of temozolomide (TMZ). The effect on cell viability was evaluated on the TMZ-resistant T98G glioma cell line for TMZ-MPSNPs, PNA-MPSNPs, and PNA-TMZ-MPSNPs. The comparative analysis showed that the synergistic effect of PNA-TMZ-MPSNPs led to a much higher increase of induced apoptosis compared to the values observed when the cancer cells were separately treated with PNA-MPSNPs and TMZ-MPSNPs, reporting a total of 70.9% of apoptotic cells using 0.5 mg mL^−1^ of the co-loaded vehicles. Noteworthy, temozolomide-resistant cells were found to become more susceptible to TMZ when anti-miR221 PNA was co-administered using these nanocarriers [181]. 

Zaho and coworkers demonstrated that a redox-triggered intracellular release of Cy5-labeled antisense PNA was an effective strategy to achieve a controlled drug release and the subsequent silencing of the B-cell lymphoma 2 (Bcl-2) protein. To this end, a sequence-specific Cy5-PNA (Bcl-2) was linked to fluorescein isothiocyanate-labeled MSNPs (MSNP-FITC) previously functionalized with a disulfide bond. The release was monitored by confocal laser scanning microscopy and it was observed that in the presence of a natural reducing agent (i.e., glutathione) inside HeLa cells, the disulfide bond was cleaved leading to Cy5-PNA liberation into the cytoplasm. It is also worth mentioning that in the absence of disulfide bond the release of Cy5-PNA was not observed and, in addition, the use of free Cy5-PNA at high concentration showed a low cellular uptake, thus confirming the efficacy of the PNA delivery through MPSNPs. Lastly, the Bcl-2 protein expression in HeLa cell line was semi-quantified by Western blot assay, which showed a decreasing in the band intensity related to the protein expression upon increased concentration of MSNP-Cy5-PNA (Bcl-2) [182].

This approach is very promising since new versions of breakable MSNP, which are degraded by external stimuli, are now available, thus allowing for targeted delivery and elimination of the nanocarrier after the carriage of active components [183,184].

### 5.3. Porous Silicon

**Figure 7:**
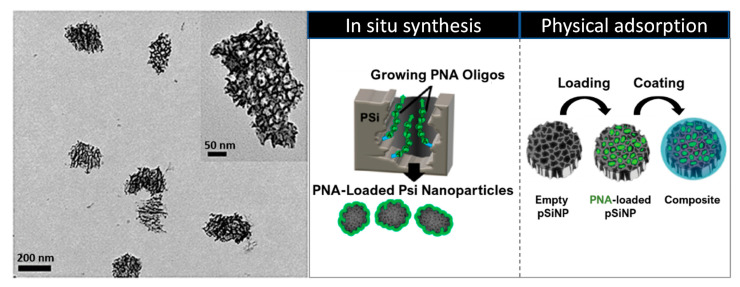
Left panel: transmission electron microscope (TEM) image of unmodified pSiNPs; the inset shows a closer view of a single nanoparticle. Right panel: in situ synthesized PNA (**left**); physically adsorbed PNA (**right**). Adapted with permission from [187,188,189]: Bertucci et al. © 2019 American Chemical Society; Beavers et al. © 2014 American Chemical Society; Beavers et al. © 2016 WILEY-VCH Verlag GmbH and Co., KGaA, Weinheim.

Among the nanocarriers used in medicine, a very interesting example is based on porous silicon nanoparticles (Figure 7), which have been shown to be stable enough to allow systemic delivery. Over the years, porous silicon nanomaterials (pSi) have shown to be suitable tools for biomedical applications as much as MSNPs. Canham and co-workers demonstrated their efficient visible photoluminescence and biocompatibility in a simulated body fluid in the mid-1990s [185,186]. Electrochemical anodization of single crystalline Si wafers in hydrofluoric acid solution is the most common procedure to fabricate pSi materials, whose morphological properties, such as pore size, porosity, and thickness, can be adjusted by varying the current density, HF concentration, etching time, and wafer type, and crystalline orientation. The surface of freshly etched pSi contains hydrophobic Si hydrides (SiH, SiH_2_, SiH_3_) that are subject to oxidation over time in ambient conditions, thereby affecting the structural and optical properties of the system as well as its degradation kinetics in aqueous media. Hence, the surface modification with various chemical functionalities plays a major role in both stabilizing the pSi layer and tuning the dissolution rate of the nanomaterial. The ease in which pSi pore geometry can be tuned during the fabrication process makes porous Si materials attractive and useful tools for drug delivery applications, and a number of papers have been published in this respect starting from the year 2000.

As mentioned above, interesting properties of these materials are their biodegradability at a suitable rate to allow release of cargo without leaving undesired debris in tissues and cells, together with their intrinsic luminescent properties [190]. These nanoparticles, appropriately coated, can be used for targeting specific tissues or organs, and have been recently used to deliver siRNA [191] and to treat *Pseudomonas aeruginosa* infections in lungs [192].

A first example of a PNA-pSi platform for applications in gene therapy and biosensing was reported by Duvall et al. in 2014 [188]. PNA synthesis within pSi was performed, using a sequence designed to inhibit miR-122, a liver-specific miRNA largely expressed in hepatocytes and involved in cholesterol metabolism and liver function. By measuring the optical thickness of the loaded pSi film, they found that the loading capacity achieved by using in situ synthesis was 8-fold higher than the one obtained with the conjugation of pre-synthesized PNA, although it was comparable in the case of a physical adsorption within the pores. Next, the release profiles of physically-adsorbed and in situ synthesized PNA were compared, proving that the covalent connection provided by in situ synthesis leads to more sustained release profile. In the light of these findings, the anti-miR activity, cellular uptake and cytotoxicity of PNA-pSi were evaluated in Huh7 (human hepatic carcinoma) cells, confirming the potential of this platform to enhance both the intracellular delivery and the bioactivity of therapeutics. Additionally, in view of biosensing applications, the sequence selectivity of an in situ synthesized 16-mer PNA on pSi biosensors towards a full complementary DNA target was established. 

In a following work [189], with the aim to facilitate the endosomal escape of PNA-pSiNPs delivery platforms, the same authors developed an endosomolytic poly[(ethylene glycol)-*block*-(dimethylamino)ethylmethacrylate-co-butylmethacrylate)] polymer (PEG-DB) able to disrupt membranes in acidic environments. For this scope, anti-miR-122 PNA was loaded within pSiNPs by physical adsorption and, subsequently, the particles were coated electrostatically with the polymer PEG-DB. From preliminary spectroscopic evaluations, the nanocomposite system showed a higher colloidal stability in physiological conditions compared to the uncoated pSiNPs, thus, cellular uptake and anti-miRNA activity were evaluated using Huh7 human liver cancer cells. The studies revealed that despite the PNA uptake being lower in the case of coated nanoparticles, the latter improved the cytosolic delivery, miRNA inhibitory activity (10-fold higher), the blood circulation half-life of anti-miR122 PNA and, as a result, its bioavailability. Additionally, subsequent in vivo investigations performed on female C57BL/6J mice have not only confirmed the results previously obtained (46% reduction in miR-122 levels relative to the empty carrier control), but have also shown that the treatment with nanocomposite particles did not cause liver or kidney toxicity. 

The same group has recently reported an interesting study regarding the effect of PEG-DB diblock-copolymer composition on serum stability, endosome escape and bioavailability for the nanocomposite system mentioned above [193]. The endosomolytic polymer is formed by the PEG block that contributes to colloidal stability of the nanoparticles, and by the two D ((dimethylamino)ethyl methacrylate) and B (butyl methacrylate) monomers, which are implied in the pH-responsive function. Since it has been assumed that the efficiency of endosome escape is higher in early endosomal vesicles (pH = 6.8) [194], various PEG-DB compositions (specifically increasing the percentage of hydrophobic B content from 20% to 70%) have been screened at different pH values in order to evaluate whether a robust membrane disruption at pH 6.8 correlates with the intracellular PNA bioactivity. In this study, they first observed that the stability of the polymer coating increases with the hydrophobicity (% B), which also drives the lipid bilayer membrane insertion. Next, it was found that the best balance between serum stability at physiological pH (7.4), pH-activated membrane disruption and a high bioactivity was provided by 40% B pSiNP composite. Indeed, although 30%B pSiNP composite showed a robust endosome disruption at the desired pH and the strongest bioavailability of the series, it also exhibited a premature release of the polymer at pH 7.4 that could cause hemotoxicity.

### 5.4. Miscellaneous Inorganic Nanocarriers

In addition to silica- and silicon-based carriers, other nanostructured inorganic materials have been used for PNA delivery. In a recent work, 2D Mg-Al layered double hydroxides (LDHs) were used in combination with a PNA targeting the KRAS G12D mutated gene, and the inhibition of pancreatic cancer was evaluated in cells and in xenografted mice [195]. This work is interesting since the ability of PNA to discriminate between mutated and wild type DNA was exploited. A proton sponge effect exerted by the nanoparticles was found to be occurring, promoting the release of the PNA from endosomes. Multifunctional magnetic nanoparticles (MNPs) have been widely employed in nanomedicine, e.g., for heat-triggered drug release or hyperthermia therapy and diagnosis. The conjugation of PNA strands to MNPs [196] and their binding to DNA have been studied [197], resulting a promising tools for targeted therapy. Interaction of PNAs with gold nanoparticles was recently studied to produce a delivery system for virus-infected cells, leading to the presence of reduced levels of the viral RNA of interest [198]. 

## 6. Carbon-Based Nanocarriers

**Figure 8:**
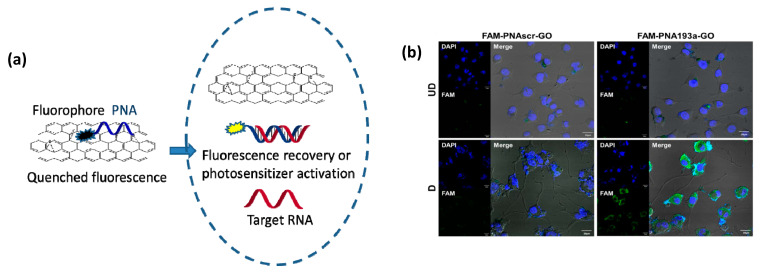
(**a**) Schematic representation of mRNA sensing or photosensitizer activation by a GO platform functionalized with a fluorophore-labelled PNA; (**b**) Detection of miR-193a in UD- or D-F11 cells after treatment with GO complexes including a fluorescein labelled-antimiR-193 PNA (FAM-PNA193a-GO) or a fluorescein labelled-scrambled PNA (FAM-PNAscr-GO). Scale bar, 20 µm. UD: undifferentiated cells, D: differentiated cells. Panel (**b**) is reprinted with permission from [201]: Oh et al. © 2018 Elsevier B.V.

PNAs can be loaded onto carbon nanostructures using non-covalent interaction, namely stacking interactions of the nucleobases or of aromatic moieties conjugated to these oligomers. The most important studies in this field reported the development of systems exploiting the interactions of PNA with graphene oxide (Figure 8) for sensing applications [199,200]. PNA-modified field effect transistors were obtained using stacking interactions of a pyrene residue conjugated to the PNA chain. Fluorescently-labelled PNAs were used in a series of studies in combination with graphene and graphene oxide. Stacking of the aromatic moieties of the fluorophore onto the graphene layer causes a complete quenching of fluorescence. The interaction with complementary DNA or RNA with PNA, which is highly sequence selective, induces the formation of a hydrophilic duplex due to the presence of charges on the nucleic acid counterpart, leading to the dissociation of the adduct from the graphene layer to restore the fluorescence emission. 

An important finding made by Min and co-workers was that nanographene oxide (nGO) loaded with a fluorescent PNAs can be efficiently delivered into eukaryotic cells [202]. Thus, the nGO moiety acts as a carrier for cellular delivery and, at the same time, as a very efficient quencher. This allowed to develop a powerful and simple method for assessing the levels of miRNA expression in living cells and, in a subsequent work, this method enabled to even visualize miRNA transfer through exosomes [201]. This approach allows to avoid RT-PCR analysis and directly perform quantification at the single cell level, with in addition temporal scans showing the evolution of the system due to external stimuli or cell changes. 

A simplified procedure based on this graphene oxide-PNA system for fluorescence in situ hybridization (G-FISH) assays in tissue was recently developed, allowing to detect the housekeeping β-actin mRNA as a control and target non-coding RNA BC1, miR-21 and miR-124 in cultured MDA-MB-231 cells (breast cancer), in different deparaffinized FFPE, in frozen tissues, and in transparent hydrogels (Clarity) specimens. The results paralleled those obtained with RT-PCR [203].

The recovery of fluorescence emission is a sign of the enhanced excited state lifetime of the fluorophore, and this effect can also be used to restore the activity of a photosensitizer. Thus, a PNA conjugated with chlorine E6 photosensitizer and complementary to miR-21—a microRNA overexpressed in tumor cells—was used in combination with dextran-coated reduced nGO to selectively target cancer cells by phototherapy. The binding of PNA to miR-21 induced a separation of the PNA from nGO, thus restoring both fluorescence and photosensitizing activity of Ce6, which produces singlet oxygen upon near IR irradiation; thus, MDA-MB-231 cells overexpressing the target miRNA underwent selective damage and severely reduced viability. The same effect was subsequently demonstrated in xenograft mice models, where the tumor mass was massively reduced after a single selective photodynamic therapy treatment [204]. 

PEG-grafted nanographene oxide (PEG-nGO) was used by Kim and co-workers to deliver PNA into cells, showing that the uptake by lung cancer cells occurred via endocytosis, ultimately down-regulating the target gene [205]. The PEG-modified nGO was found to be more efficient in delivery than pristine nGO under the same conditions. Most importantly, it was demonstrated that, unlike PEG-nGO itself which is localized into endosomal compartments and then in lysosomes, a fluorescently labelled PNA could diffuse in the cytoplasm, due to efficient endosomal escape.

In a recent work, coating of the GO sheets with hyaluronic acids has been performed for delivering a Cy3-fluorescently-labelled PNA targeted to the oncogenic miR-21 into CD44-positive MBA-MB231 cells. The binding of PNA to the target miR-21 produced both a fluorescent signal proportional to the level of target RNA and a therapeutic effect by blocking the miR activity, decreasing proliferation and migration, and increasing apoptosis [206]. This is an important demonstration that theranostics, i.e., combined therapeutic and diagnosis functions at the same time, can be performed with PNA-based multifunctional systems.

Other carbon-based nanomaterials were used for PNA delivery. Carbon nitride nanosheets (CNNS) were used with a strategy similar to that exploited for nGO [207]; the nanosheets were loaded with a miR-18a-targeting PNA-Cy5 conjugate and delivery to cells was evaluated. Adsorption of folate-conjugated Poly-A on the same sheets allowed to selectively deliver this multifunctional system to hepatocytes through receptor-mediated endocytosis and to target cytoplasm compartment.

PNAs were also directly conjugated to nanodiamonds (ND) by Arnaud and co-workers, and the PNA-ND conjugates were shown to selectively interact with DNA and to be carried into A549 cells without toxicity, though no biological activity was tested [208]. This preliminary report is promising, since nanodiamonds have unique optical and magnetic properties.

Oxidized carbon nanoparticles, prepared from graphite, can deliver macromolecules into cells, and were so far used to transport PNA analogs (acpcPNA, i.e., bearing a D-prolyl-(1*S*,2*S*)-2-aminocyclopentanecarboxylic acid backbone) inside the nucleus of cells, and produced anti-gene effects blocking the transcription of interleukin-6 (IL6) mRNA and protein [209].

## 7. Supramolecular Multifunctional Systems

### 7.1. Peptide, Protein, and PNA Nanoparticles

Recently, amphipathic peptides, cell penetrating peptides conjugates and peptide-liposome complexes were used for the realization of self-assembled nanostructures [210]. Similarly to the self-assembled lipids- and phospholipids-based systems reported in Section 5, peptide and protein constructs can be used to transfect nucleic acids or proteins within cells. For example, SV40 Pseudovirion particles, formed when the SV40 capsid protein (VP1) self-assembles around nucleic acids, were found to be active as transfectants for PNAs at 10 μM concentration in cell cultures, inducing transcription block of MDM1 gene and reduction of multi-drug resistance [211]. 

A similar approach based on self-assembled Pep-1 (Chariot) peptide was shown to internalize PNAs, but the available data report only the case of PNA phosphonates (PHONA) [212]. A new generation of this type of peptide has been selected (Pep-2), enabling transfection of PNAs and modified PNAs directed against Cyclin B1. The transfection allowed to inhibit both the gene and cell cycle progression, and tumor cell proliferation [213].

In these cases, it is clear that peptide and protein assemblies are able on one side to cross the cell membrane as described for other cargo molecules, and on the other side to interact with the tested PNAs by non-covalent interactions. These interactions are not precisely identified; it is, however, very interesting to consider that PNA-protein association could be decisive also in other still unexplored contexts. One recent report on this problem, carried out by Winssinger and co-workers, allowed to identify protein-PNA interactions that were dependent on the backbone and on the sequence of the given oligomers, and to pinpoint a particular protein (Caprin-1) that was shown to co-assemble with both γ-serine- and α-arginine-modified PNAs by capture experiments and to mediate their intracellular delivery [214]. 

Interestingly, Nielsen and co-workers found that PNA uptake can be enhanced by the formation of nanoaggregates. This was first observed by using PNA-oligo(bicycloguanidinium) conjugates, which were shown to aggregate into nanostructures [215]. In a subsequent work, they found that poly-arginine-conjugated PNAs of unrelated sequence could enhance the antisense activity of another antisense PNA, reporting a more pronounced effect for oligomers containing a lipophilic moiety. This correlated with the formation of PNA nanoparticles that acted as carrier for all the PNA components. These nanoparticles were found to be formed in the presence of serum proteins, which were effectively detected in the nanoparticle pellet [216]. 

### 7.2. Cationic Calixarenes

**Figure 9:**
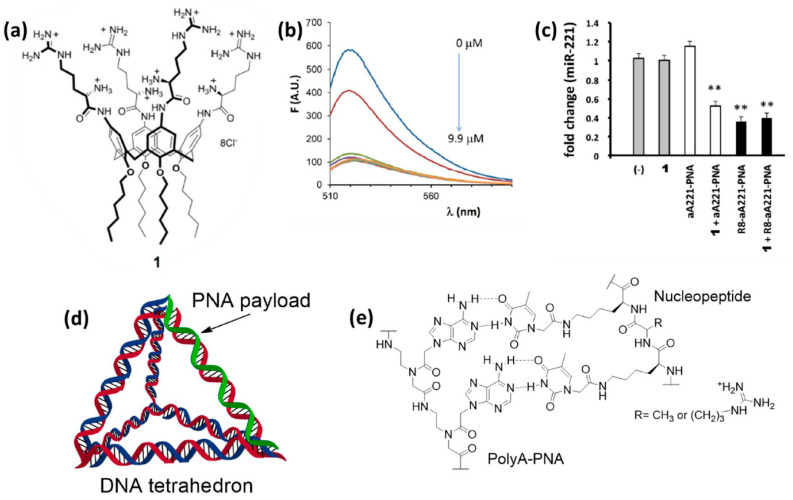
Supramolecular carriers. (**a**) Structure of the arginine-based calix[4]arene **1** used in the transfection of PNAs; (**b**) fluorescence titration of Fluorescein-labelled PNAa221 directed against miR-221 (a221) by addition of increasing concentrations of calixarene **1** (from top to bottom: 0, 1.0, 2.0, 2.9, 3.9, 4.8, 5.7, 6.5, 7.4, 8.3, 9.1, 9.9 µM); (**c**) anti-miR 221 effect of transfected PNA a221 or its variant with octa-arginine CPP (R8-a221-PNA) in glioma U251 cells, in the absence and in the presence of calixarene **1**, ** *p* < 0.01; (**d**) DNA tetrahedron for the delivery of PNA payloads in bacterial cells; (**e**) section of a complex containing a polyA-PNA and a thymine-modified nucleopeptide tested for the transfection of the former component in ref. [220]. Panels (**a**–**c**) are reprinted from [219]: Gasparello et al. © 2019 Creative Commons Attribution 4.0 International License.

It is clear that the protein-PNA interactions shown in the systems reported above should rely on the multivalency effect, with the presence of multiple non-covalent binding sites. One similar case, but in a more simplified form, was observed for multivalent carriers based on calixarenes. Ammonium- and guanidium-based calix[4]arenes with lipophilic tails were developed by Casnati, Sansone, and coworkers as efficient transfecting agents for DNA and RNA, allowing DNA condensation [217] and miR delivery [218]. It was originally postulated that electrostatic interactions with the guanidinium and ammonium residues was decisive for the interaction with nucleic acids and transfection. However, these derivatives were shown to be able to transfect also anti-miR PNAs into glioma cells, obtaining the best results in the presence of compound **1** (Figure 9a) [219]. A fluorescently-labelled PNA was found to interact with the calixarene carrier by measuring the quenching of fluorescence resulting from its association with the supramolecular derivative, thus showing that the calixarene was in effect carrying the PNA and simply permeabilizing cells (Figure 9b). From this study was thus evident that PNAs can interact with amino acidic residues, and, in particular, that a combination of amino and guanidino groups was effective in promoting these interactions and to enhance the calixarene-mediated transfection. Targeting of miR-221, overexpressed in many tumors, was performed in glioma U251 cells. The transfection of unconjugated (naked) anti-miR-221 PNA (a221-PNA) was found to be efficient as evaluated by cellular uptake studies and miR bioavailability assays (Figure 9c). On the other hand, the combination of a221-PNA and **1** was found to induce higher apoptosis than the bare a221-PNA and the corresponding octa-arginine conjugate (R8-a221-PNA), although the latter was found to have similar anti-miR effect. One practical point of using these carriers is the simplicity of preparation by bare carrier/PNA mixing procedures. Another interesting aspect of this system is that it provided evidence of the occurrence of intermolecular interactions of the neutral PNA with the carrier, and in particular with the positively-charged ammonium and guanidinium groups.

### 7.3. DNA Nanostructures and Nucleopeptides

DNA nanotechnology exploits the predictable and programmable self-assembly of DNA oligonucleotides for creating nanoscaled, non-canonical architectures with rational shape and dimension [221]. Over the years, different strategies for building multifunctional scaffolds have been explored, leading to the creation of highly sophisticated systems which are able to respond to external stimuli and to undergo further arrangement in higher order structures [222,223]. Moreover, the development of valid approaches for the post-assembly engineering with various (macro)molecular entities have allowed the creation of interesting platforms for bioimaging, biosensing, and drug delivery [221,224]. Regarding the transport of PNA derivatives, an effective scaffold was obtained by designing simple DNA aggregates for clustering an increasing number of CPP-modified PNAs, thus creating a multivalent system with improved uptake properties [225]. More in detail, the sequences forming the carrier were composed of a central part able to form a duplex or a triplex with other DNA oligonucleotides, flanked by two toeholds for anchoring antisense or antigene PNAs. For the central segment of the DNA strands 12-T, 12-G, 12-A, 12-C, and 12-mer mixed sequences were tested for exploring the formation of DNA complexes with different stability and multiplicity, while the PNA cargoes were distinguished by the peptide attached on their C-term. When the carried PNA was expected to perform antisense activity against the hepatitis B virus (HBV) genome in HepG2 cells, the best results were obtained when polyC and polyG DNAs were used to build the nanovehicles and NLS (nuclear localization sequence peptide) was used as CPP on the PNA. In fact, the 12-C and 12-G sequences, together with forming a more stable scaffold thanks to the stronger pairing between the nucleobases, can give rise to a triplex structure able to accommodate six PNA strands instead of the four that can be transported with a double stranded platform. The multivalent system was taken up by cells at least 10-fold more efficiently than a simple NLS-PNA molecule, despite it being reported to be mainly localized in endosomes. To solve this problem, a PNA bearing a polyhistidine chain was co-assembled on the hybrid carrier, leading to an improvement of the antisense activity of the system thanks to the endosomolytic action of the additional CPP [226]. The same scaffold was also effective in the delivery of different CPP-PNAs possessing antigene sequences, correctly targeting the planned sites of episomal viral DNA or transfected plasmids in the tested cell cultures. Additionally, in these cases, a cooperation between co-loaded polyhistidine- and CPP-modified PNAs was reported, with the former derivative providing an enhanced endosomal escape for the latter to display a higher antigene activity.

A more advanced strategy for the delivery of PNAs consisted in the use of a DNA tetrahedron (DNA-Td) for the transfection of bacterial cells (Figure 9d). DNA-Tds can be obtained from four single stranded oligomers by designing their sequences to be complementary with appropriate regions of other strands, in order to obtain the planned 3D architecture by self-assembling the components according to the Watson–Crick base pairing rules [221]. When one of the DNA strand is shorter than the others, the resulting tetrahedron will have a single stranded region on one of its borders, giving the possibility to functionalize that site with additional oligonucleotides, such as PNAs. A hybrid PNA/DNA Td (PT01) was used to inhibit the translation of the ®-lactamase CTX-M-group 1 gene in *Escherichia Coli* (*E. Coli*) [227], to suppress their resistance to the cefotaxime (CTX) antibiotic [228]. Co-incubation of LREC461 *E. Coli* with PT01 and CTX yielded a dose-dependent inhibition of bacterial growth which was associated to the synergistic effect of the antibiotic and the antisense PNA, without reporting toxic effects upon administration of the naked DNA-Td. Hence, it was supposed that the PT01 complex was able to release its PNA cargo by dissociation of the PNA/DNA duplex forming one of its border, probably facilitated by the degradation of the DNA component by endonuclease enzymes. A similar approach was used in another study to treat methicillin-resistant *Staphylococcus aureus* (MRSA) [229], but in this case an antisense PNA was used as an alternative of antibiotic agents rather than as a support. In fact, a DNA-Td was paired with a PNA targeting the *fts*Z gene (*fts*Z-PNA), whose suppression represent an interesting strategy for the development of antimicrobial treatment as it is involved in bacterial cell division, but it is not expressed by animal cells [230]. MRSA cultured with the hybrid vector experienced a suppression of their growth in a dose-dependent manner, while incubation with *fts*Z-PNA alone did not affect significantly bacterial growth. These observations confirmed the transfection activity of the DNA tetrahedron, while the antisense function of the PNA component was confirmed by a combination of PCR analyses. In fact, the incubation of MRSA with increasing concentrations of the PNA-modified Td resulted in a proportional decrease of the *fts*Z expression without affecting other genes, thus validating the direct and selective action of *fts*Z-PNA against drug-resistant bacteria.

Nucleopeptides (Figure 9e) are a family of artificial macromolecules that have been used for cellular delivery purposes [220,231]. These oligomers are peptide mimics bearing nucleobases on some of their side chains, thus allowing the interaction with complementary nucleic acids. Moreover, when cationic amino acids are selected as unmodified residues, they can improve the cell internalization of the complexed oligonucleotides, thus simulating the action of CPPs. Accordingly, DNA, RNA, and PNA strands have been reported to cross cell membranes with the help of these particular partners, in the absence of other transfecting agents. Focusing on PNAs, it has been shown that a hexa-adenine derivative can be carried into HT29 cells by a 12-mer nucleopeptide alternating a varying number of alanine or arginine residues with six lysines presenting thymine moieties on their lateral groups [220]. Interestingly, the best delivery performance was observed for a nucleopeptide bearing only two positive charges (two Arg residues) on its backbone, are contrary to what was observed for CPPs that, despite their similar structure, require a high number of cationic groups to work as transport mediators.

## 8. Conclusions

The reader of PNA-related literature is often encounters some sentences stating that PNAs have “poor cellular uptake”, which is almost unanimously considered as the major problem in the development of PNA-based treatments. From the overview presented here it is clear that, in recent years, great advances have been made in the direction of making PNAs effective in reaching their target. In fact, functional systems enabling not only crossing the cellular membrane or cell wall, but also to manipulate intracellular trafficking, are now available. Most importantly, new functions, such as genome editing, targeted delivery, or theranostics, have been performed thanks to the unique properties of PNAs. In particular, the availability of synthetic methods for manipulating the charges, conformation, and handedness of PNAs, together with their robustness and both their chemical and enzymatic stability, make these oligomers an ideal component of engineered nanosystems for biological applications. The combination of PNAs with new nanomaterials has been successful in many cases, and it is increasingly based on the capability to control non-covalent interactions. Still, some aspects of the interactions of PNAs with the biological environment, in particular their self-assembly and their interactions with proteins or carriers, are only partly understood and the complete picture of their interactome will probably be a leading subject in the next developments of PNA-based technology.

## References

[1] Nielsen P., Egholm M., Berg R., Buchardt O. (1991). Sequence-selective recognition of DNA by strand displacement with a thymine-substituted polyamide. Science.

[2] Egholm M., Buchardt O., Christensen L., Behrens C., Freier S.M., Driver D.A., Berg R.H., Kim S.K., Norden B., Nielsen P.E. (1993). PNA hybridizes to complementary oligonucleotides obeying the Watson–Crick hydrogen-bonding rules. Nat. Cell Biol..

[3] Brown S.C., A Thomson S., Veal J.M., Davis D.G. (1994). NMR solution structure of a peptide nucleic acid complexed with RNA. Science.

[4] Wittung P., Nielsen P., Nordén B., Wittung-Stafshede P. (1996). Direct Observation of Strand Invasion by Peptide Nucleic Acid (PNA) into Double-Stranded DNA. J. Am. Chem. Soc..

[5] Nielsen P.E. (2001). Targeting Double Stranded DNA with Peptide Nucleic Acid (PNA). Curr. Med. Chem..

[6] Saarbach J., Sabale P.M., Winssinger N. (2019). Peptide nucleic acid (PNA) and its applications in chemical biology, diagnostics, and therapeutics. Curr. Opin. Chem. Biol..

[7] Nielsen P.E. (2010). Gene Targeting and Expression Modulation by Peptide Nucleic Acids (PNA). Curr. Pharm. Des..

[8] Nielsen P.E. (2010). Peptide Nucleic Acids (PNA) in Chemical Biology and Drug Discovery. Chem. Biodivers..

[9] Dong B., Nie K., Shi H., Chao L., Ma M., Gao F., Liang B., Chen W., Long M., Liu Z. (2019). Film-Spotting chiral miniPEG-γPNA array for BRCA1 gene mutation detection. Biosens. Bioelectron..

[10] Zhang N., Appella D.H. (2007). Colorimetric Detection of Anthrax DNA with a Peptide Nucleic Acid Sandwich-Hybridization Assay. J. Am. Chem. Soc..

[11] D’Agata R., Bellassai N., Allegretti M., Rozzi A., Korom S., Manicardi A., Melucci E., Pescarmona E., Corradini R., Giacomini P. (2020). Direct plasmonic detection of circulating RAS mutated DNA in colorectal cancer patients. Biosens. Bioelectron..

[12] Kumar S., Pearse A., Liu Y., Taylor R.E. (2020). Modular self-assembly of gamma-modified peptide nucleic acids in organic solvent mixtures. Nat. Commun..

[13] Swenson C.S., Heemstra J.M. (2020). Peptide nucleic acids harness dual information codes in a single molecule. Chem. Commun..

[14] Totsingan F., Marchelli R., Corradini R. (2011). Molecular Computing by PNA. Artif. DNA PNA XNA.

[15] Demidov V.V., Potaman V.N., Frank-Kamenetskil M., Egholm M., Buchard O., Sönnichsen S.H., Nlelsen P.E. (1994). Stability of peptide nucleic acids in human serum and cellular extracts. Biochem. Pharmacol..

[16] Wittung P., Nielsen P.E., Buchardt O., Egholm M., Norde B. (1994). DNA-like double helix formed by peptide nucleic acid. Nat. Cell Biol..

[17] D’Agata R., Giuffrida M.C., Spoto G. (2017). Peptide Nucleic Acid-Based Biosensors for Cancer Diagnosis. Molecules.

[18] Singh R.P., Oh B.-K., Choi J.-W. (2010). Application of peptide nucleic acid towards development of nanobiosensor arrays. Bioelectrochemistry.

[19] Tedeschi T., Sforza S., Dossena A., Corradini R., Marchelli R. (2005). Lysine-based peptide nucleic acids (PNAs) with strong chiral constraint: Control of helix handedness and DNA binding by chirality. Chirality.

[20] Sacui I., Hsieh W.-C., Manna A., Sahu B., Ly D.H. (2015). Gamma Peptide Nucleic Acids: As Orthogonal Nucleic Acid Recognition Codes for Organizing Molecular Self-Assembly. J. Am. Chem. Soc..

[21] Liu Y., Braasch D.A., Nulf C.J., Corey D.R. (2004). Efficient and Isoform-Selective Inhibition of Cellular Gene Expression by Peptide Nucleic Acids†. Biochemistry.

[22] Ivanova G.D., Arzumanov A., Abes R., Yin H., Wood M.J.A., LeBleu B., Gait M.J. (2008). Improved cell-penetrating peptide–PNA conjugates for splicing redirection in HeLa cells and exon skipping in mdx mouse muscle. Nucleic Acids Res..

[23] Yin H., Betts C.A., Saleh A.F., Ivanova G.D., Lee H., Seow Y., Kim D., Gait M.J., Wood M.J.A. (2010). Optimization of Peptide Nucleic Acid Antisense Oligonucleotides for Local and Systemic Dystrophin Splice Correction in the mdx Mouse. Mol. Ther..

[24] Lee H.T., Kim S.K., Yoon J.W. (2019). Antisense peptide nucleic acids as a potential anti-infective agent. J. Microbiol..

[25] Ghosal A., Nielsen P.E. (2012). Potent Antibacterial Antisense Peptide–Peptide Nucleic Acid Conjugates Against Pseudomonas aeruginosa. Nucleic Acid Ther..

[26] Good L., Stach J.E.M. (2011). Synthetic RNA Silencing in Bacteria? Antimicrobial Discovery and Resistance Breaking. Front. Microbiol..

[27] Cutrona G., Carpaneto E.M., Ulivi M., Roncella S., Landt O., Ferrarini M., Boffa L.C. (2000). Effects in live cells of a c-myc anti-gene PNA linked to a nuclear localization signal. Nat. Biotechnol..

[28] Tonelli R., Purgato S., Camerin C., Fronza R., Bologna F., Alboresi S., Franzoni M., Corradini R., Sforza S., Faccini A. (2005). Anti-gene peptide nucleic acid specifically inhibits MYCN expression in human neuroblastoma cells leading to cell growth inhibition and apoptosis. Mol. Cancer Ther..

[29] Hu J., Corey D.R. (2007). Inhibiting Gene Expression with Peptide Nucleic Acid (PNA)−Peptide Conjugates That Target Chromosomal DNA†. Biochemistry.

[30] Fabani M.M., Abreu-Goodger C., Williams D., Lyons P.A., Torres A.G., Smith K.G.C., Enright A.J., Gait M.J., Vigorito E. (2010). Efficient inhibition of miR-155 function in vivo by peptide nucleic acids. Nucleic Acids Res..

[31] Cheng C.J., Bahal R., Babar I.A., Pincus Z., Barrera F.N., Liu C., A Svoronos A., Braddock D.T., Glazer P.M., Engelman D.M. (2015). MicroRNA silencing for cancer therapy targeted to the tumour microenvironment. Nat. Cell Biol..

[32] Gambari R., Fabbri E., Borgatti M., Lampronti I., Finotti A., Brognara E., Bianchi N., Manicardi A., Marchelli R., Corradini R. (2011). Targeting microRNAs involved in human diseases: A novel approach for modification of gene expression and drug development. Biochem. Pharmacol..

[33] Fabani M.M., Gait M.J. (2007). miR-122 targeting with LNA/2’-O-methyl oligonucleotide mixmers, peptide nucleic acids (PNA), and PNA-peptide conjugates. RNA.

[34] Borgatti M., Lampronti I., Romanelli A., Pedone C., Saviano M., Bianchi N., Mischiati C., Gambari R. (2002). Transcription Factor Decoy Molecules Based on a Peptide Nucleic Acid (PNA)-DNA Chimera Mimicking Sp1 Binding Sites. J. Biol. Chem..

[35] Knudsen H., Nielsen P.E. (1996). Antisense Properties of Duplex- and Triplex-Forming PNAs. Nucleic Acids Res..

[36] Hanvey J.C., Peffer N.J., E Bisi J., A Thomson S., Cadilla R., A Josey J., Ricca D.J., Hassman C.F., A Bonham M., Au K.G. (1992). Antisense and antigene properties of peptide nucleic acids. Science.

[37] Gupta A., Mishra A., Puri N. (2017). Peptide nucleic acids: Advanced tools for biomedical applications. J. Biotechnol..

[38] Economos N.G., Oyaghire S., Quijano E., Ricciardi A.S., Saltzman W.M., Glazer P.M. (2020). Peptide Nucleic Acids and Gene Editing: Perspectives on Structure and Repair. Molecules.

[39] Koppelhus U., E Nielsen P. (2003). Cellular delivery of peptide nucleic acid (PNA). Adv. Drug Deliv. Rev..

[40] Gupta A., Bahal R., Gupta M., Glazer P.M., Saltzman W.M. (2016). Nanotechnology for delivery of peptide nucleic acids (PNAs). J. Control. Release.

[41] Malik S., Asmara B., Moscato Z., Mukker J.K., Bahal R. (2019). Advances in Nanoparticle-based Delivery of Next Generation Peptide Nucleic Acids. Curr. Pharm. Des..

[42] Manicardi A., Rozzi A., Korom S., Corradini R. (2017). Building on the peptide nucleic acid (PNA) scaffold: A biomolecular engineering approach. Supramol. Chem..

[43] Adlerz L., Soomets U., Holmlund L., Viirlaid S., Langel Ü., Iverfeldt K. (2003). Down-regulation of amyloid precursor protein by peptide nucleic acid oligomer in cultured rat primary neurons and astrocytes. Neurosci. Lett..

[44] Good L., Sandberg R., Larsson O., Nielsen P.E., Wahlestedt C. (2000). Antisense PNA effects in Escherichia coli are limited by the outer-membrane LPS layer. Microbiology.

[45] Hirschman S.Z., Chen C.W. (1996). Peptide nucleic acids stimulate gamma interferon and inhibit the replication of the human immunodeficiency virus. J. Investig. Med..

[46] Wang G., Xu X., Pace B., Dean D.A., Glazer P.M., Chan P., Goodman S.R., Shokolenko I. (1999). Peptide nucleic acid (PNA) binding-mediated induction of human-globin gene expression. Nucleic Acids Res..

[47] Doyle D.F., Braasch D.A., Janowski B.A., Corey D.R. (2001). Inhibition of Gene Expression Inside Cells by Peptide Nucleic Acids: Effect of mRNA Target Sequence, Mismatched Bases, and PNA Length. Biochemistry.

[48] Faruqi A.F., Egholm M., Glazer P.M. (1998). Peptide nucleic acid-targeted mutagenesis of a chromosomal gene in mouse cells. Proc. Natl. Acad. Sci. USA.

[49] Mitra R., Ganesh K.N. (2012). Aminomethylene Peptide Nucleic Acid (am-PNA): Synthesis, Regio-/Stereospecific DNA Binding, And Differential Cell Uptake of (α/γ,R/S)am-PNA Analogues. J. Org. Chem..

[50] Kumar P., Jain D.R. (2015). Cγ-Aminopropylene peptide nucleic acid (amp-PNA): Chiral cationic PNAs with superior PNA:DNA/RNA duplex stability and cellular uptake. Tetrahedron.

[51] Delgado E., Bahal R., Yang J., Lee J.M., Ly D.H., Monga S.P. (2013). β-Catenin Knockdown in Liver Tumor Cells by a Cell Permeable Gamma Guanidine-based Peptide Nucleic Acid. Curr. Cancer Drug Targets.

[52] Dragulescu-Andrasi A., Rapireddy S., He G., Bhattacharya B., Hyldig-Nielsen J.J., Zon G., Ly D.H. (2006). Cell-Permeable Peptide Nucleic Acid Designed to Bind to the 5‘-Untranslated Region of E-cadherin Transcript Induces Potent and Sequence-Specific Antisense Effects. J. Am. Chem. Soc..

[53] Thomas S.M., Sahu B., Rapireddy S., Bahal R., Wheeler S.E., Procopio E.M., Kim J., Joyce S.C., Contrucci S., Wang Y. (2012). Antitumor Effects of EGFR Antisense Guanidine-Based Peptide Nucleic Acids in Cancer Models. ACS Chem. Biol..

[54] Zhou P., Wang M., Du L., Fisher G.W., Waggoner A., Ly D.H. (2003). Novel Binding and Efficient Cellular Uptake of Guanidine-Based Peptide Nucleic Acids (GPNA). J. Am. Chem. Soc..

[55] Sahu B., Sacui I., Chenna V., Lathrop K.L., Thomas S.M., Zon G., Livak K.J., Ly D.H. (2009). Synthesis of Conformationally Preorganized and Cell-Permeable Guanidine-Based gamma-Peptide Nucleic Acids (gamma GPNAs). J. Org. Chem..

[56] Sforza S., Tedeschi T., Corradini R., Marchelli R. (2007). Induction of Helical Handedness and DNA Binding Properties of Peptide Nucleic Acids (PNAs) with Two Stereogenic Centres. Eur. J. Org. Chem..

[57] Sugiyama T., Kittaka A. (2012). Chiral Peptide Nucleic Acids with a Substituent in the N-(2-Aminoethy) glycine Backbone. Molecules.

[58] Corradini R., Sforza S., Tedeschi T., Totsingan F., Manicardi A., Marchelli R. (2011). Peptide Nucleic Acids with a Structurally Biased Backbone. Updated Review and Emerging Challenges. Curr. Top. Med. Chem..

[59] Uğurlu Ö., Barlas F.B., Evran S., Timur S. (2020). The cell-penetrating YopM protein-functionalized quantum dot-plasmid DNA conjugate as a novel gene delivery vector. Plasmid.

[60] Hapuarachchige S., Artemov D. (2020). Theranostic Pretargeting Drug Delivery and Imaging Platforms in Cancer Precision Medicine. Front. Oncol..

[61] Zhang Z., Liu Y., Jarreau C., Welch M.J., Taylor J.-S.A. (2013). Nucleic acid-directed self-assembly of multifunctional gold nanoparticle imaging agents. Biomater. Sci..

[62] Abes S., Williams D., Prevot P., Thierry A., Gait M.J., LeBleu B. (2006). Endosome trapping limits the efficiency of splicing correction by PNA-oligolysine conjugates. J. Control. Release.

[63] Saleh A.F., Arzumanov A., Abes R., Owen D., LeBleu B., Gait M.J. (2010). Synthesis and Splice-Redirecting Activity of Branched, Arginine-Rich Peptide Dendrimer Conjugates of Peptide Nucleic Acid Oligonucleotides. Bioconjugate Chem..

[64] Braun K., Peschke P., Pipkorn R., Lampel S., Wachsmuth M., Waldeck W., Friedrich E., Debus J. (2002). A Biological Transporter for the Delivery of Peptide Nucleic Acids (PNAs) to the Nuclear Compartment of Living Cells. J. Mol. Biol..

[65] Chen S.-S., Tu X.-Y., Xie L.-X., Xiong L.-P., Song J., Ye X.-Q. (2018). Peptide nucleic acids targeting mitochondria enhances sensitivity of lung cancer cells to chemotherapy. Am. J. Transl. Res..

[66] Lamla M., Seliger H., Kaufmann D. (2010). Differences in uptake, localization, and processing of PNAs modified by COX VIII pre-sequence peptide and by triphenylphoshonium cation into mitochondria of tumor cells. Drug Deliv..

[67] McMahon B.M., Mays D., Lipsky J., Stewart J.A., Fauq A., Richelson E. (2002). Pharmacokinetics and Tissue Distribution of a Peptide Nucleic Acid After Intravenous Administration. Antisense Nucleic Acid Drug Dev..

[68] Ren J., Shen S., Wang D., Xi Z., Guo L., Pang Z., Qian Y., Sun X., Jiang X. (2012). The targeted delivery of anticancer drugs to brain glioma by PEGylated oxidized multi-walled carbon nanotubes modified with angiopep-2. Biomaterials.

[69] Ljungstrøm T., Knudsen H., Nielsen P.E. (1999). Cellular uptake of adamantyl conjugated peptide nucleic acids. Bioconjugate Chem..

[70] Muratovska A., Lightowlers R.N., Taylor R.W., Turnbull D.M., Smith R.A.J., Wilce J.A., Martin S.T.W., Murphy M.P. (2001). Targeting peptide nucleic acid (PNA) oligomers to mitochondria within cells by conjugation to lipophilic cations: Implications for mitochondrial DNA replication, expression and disease. Nucleic Acids Res..

[71] Biessen E.A.L., Sliedregt-Bol K., Chr’T Hoen P.A., Prince P., Van Der Bilt E., Valentijn A.R.P.M., Meeuwenoord N.J., Princen H.M., Bijsterbosch M.K., Van Der Marel G.A. (2002). Design of a Targeted Peptide Nucleic Acid Prodrug to Inhibit Hepatic Human Microsomal Triglyceride Transfer Protein Expression in Hepatocytes†. Bioconjugate Chem..

[72] Van Rossenberg S.M.W., Sliedregt-Bol K.M., Prince P., Van Berkel T.J.C., Van Boom J.H., Van Der Marel G.A., Biessen E.A.L. (2003). A Targeted Peptide Nucleic Acid to Down-Regulate Mouse Microsomal Triglyceride Transfer Protein Expression in Hepatocytes. Bioconjugate Chem..

[73] Bhingardeve P., Madhanagopal B.R., Naick H., Jain P., Manoharan M., Ganesh K.N. (2020). Receptor-Specific Delivery of Peptide Nucleic Acids Conjugated to Three Sequentially Linked N-Acetyl Galactosamine Moieties into Hepatocytes. J. Org. Chem..

[74] Gabas I.M., Nielsen P.E. (2019). Effective Cellular Delivery of Antisense Peptide Nucleic Acid by Conjugation to Guanidinylated Diaminobutanoic Acid-Based Peptide Dendrons. Biomacromolecules.

[75] Równicki M., Wojciechowska M., Wierzba A.J., Czarnecki J., Bartosik D., Gryko D., Trylska J. (2017). Vitamin B12 as a carrier of peptide nucleic acid (PNA) into bacterial cells. Sci. Rep..

[76] Równicki M., Dąbrowska Z., Wojciechowska M., Wierzba A.J., Maximova K., Gryko D., Trylska J. (2019). Inhibition of Escherichia coli Growth by Vitamin B12–Peptide Nucleic Acid Conjugates. ACS Omega.

[77] Wierzba A.J., Maximova K., Wincenciuk A., Równicki M., Wojciechowska M., Nexo E., Trylska J., Gryko D. (2018). Does a Conjugation Site Affect Transport of Vitamin B 12 –Peptide Nucleic Acid Conjugates into Bacterial Cells?. Chem. A Eur. J..

[78] Shen G., Fang H., Song Y., Bielska A.A., Wang Z., Taylor J.-S.A. (2009). Phospholipid Conjugate for Intracellular Delivery of Peptide Nucleic Acids. Bioconjugate Chem..

[79] Zorko M., Langel U. (2005). Cell-penetrating peptides: Mechanism and kinetics of cargo delivery. Adv. Drug Deliv. Rev..

[80] Copolovici D.M., Langel K., Eriste E., Langel Ü. (2014). Cell-Penetrating Peptides: Design, Synthesis, and Applications. ACS Nano.

[81] Pooga M., Soomets U., Hällbrink M., Valkna A., Saar K., Rezaei K., Kahl U., Hao J.-X., Xu X.-J., Wiesenfeld-Hallin Z. (1998). Cell penetrating PNA constructs regulate galanin receptor levels and modify pain transmission in vivo. Nat. Biotechnol..

[82] Rogers F.A., Lin S.S., Hegan D.C., Krause D.S., Glazer P.M. (2012). Targeted Gene Modification of Hematopoietic Progenitor Cells in Mice Following Systemic Administration of a PNA-peptide Conjugate. Mol. Ther..

[83] Tan X., Bruchez M.P., Armitage B.A. (2018). Closing the Loop: Constraining TAT Peptide by γPNA Hairpin for Enhanced Cellular Delivery of Biomolecules. Bioconjugate Chem..

[84] Zoonens M., Reshetnyak Y.K., Engelman N.M. (2008). Bilayer Interactions of pHLIP, a Peptide that Can Deliver Drugs and Target Tumors. Biophys. J..

[85] Gait M.J., Arzumanov A.A., McClorey G., Godfrey C., Betts C., Hammond S., Wood M.J. (2019). Cell-Penetrating Peptide Conjugates of Steric Blocking Oligonucleotides as Therapeutics for Neuromuscular Diseases from a Historical Perspective to Current Prospects of Treatment. Nucleic Acid Ther..

[86] Gambari R., Gasparello J., Fabbri E., Borgatti M., Tamanini A., Finotti A., Nielsen P.E. (2020). Peptide Nucleic Acids for MicroRNA Targeting. Peptide Nucleic Acids: Methods and Protocols.

[87] Wojciechowska M., Równicki M., Mieczkowski A., Miszkiewicz J., Trylska J. (2020). Antibacterial Peptide Nucleic Acids—Facts and Perspectives. Molecules.

[88] Goltermann L., Nielsen P.E., Nielsen P.E. (2020). PNA Antisense Targeting in Bacteria: Determination of Antibacterial Activity (MIC) of PNA-Peptide Conjugates. Peptide Nucleic Acids: Methods and Protocols.

[89] Fabbri E., Tamanini A., Jakova T., Gasparello J., Manicardi A., Corradini R., Sabbioni G., Finotti A., Borgatti M., Lampronti I. (2017). A Peptide Nucleic Acid against MicroRNA miR-145-5p Enhances the Expression of the Cystic Fibrosis Transmembrane Conductance Regulator (CFTR) in Calu-3 Cells. Molecules.

[90] Brognara E., Fabbri E., Aimi F., Manicardi A., Bianchi N., Finotti A., Breveglieri G., Borgatti M., Corradini R., Marchelli R. (2012). Peptide nucleic acids targeting miR-221 modulate p27Kip1 expression in breast cancer MDA-MB-231 cells. Int. J. Oncol..

[91] Ndeboko B., Ramamurthy N., Lemamy G.J., Jamard C., Nielsen P.E., Cova L. (2017). Role of Cell-Penetrating Peptides in Intracellular Delivery of Peptide Nucleic Acids Targeting Hepadnaviral Replication. Mol. Ther. Nucleic Acids.

[92] Ndeboko B., Hantz O., Lemamy G.J., Cova L. (2018). Developments in Cell-Penetrating Peptides as Antiviral Agents and as Vehicles for Delivery of Peptide Nucleic Acid Targeting Hepadnaviral Replication Pathway. Biomolecules.

[93] Kauffman W.B., Guha S., Wimley W.C. (2018). Synthetic molecular evolution of hybrid cell penetrating peptides. Nat. Commun..

[94] Soudah T., Mogilevsky M., Karni R., Yavin E. (2017). CLIP6-PNA-Peptide Conjugates: Non-Endosomal Delivery of Splice Switching Oligonucleotides. Bioconjugate Chem..

[95] Lundin P., Johansson H., Guterstam P., Holm T., Hansen M., Langel Ü., El Andaloussi S. (2008). Distinct Uptake Routes of Cell-Penetrating Peptide Conjugates. Bioconjugate Chem..

[96] Nielsen P.E. (2005). Addressing the challenges of cellular delivery and bioavailability of peptide nucleic acids (PNA). Q. Rev. Biophys..

[97] Fischer R., Brock R., Fotin-Mleczek M. (2005). Endocytosis and Cationic Cell-Penetrating Peptides—A Merger of Concepts and Methods. Curr. Pharm. Des..

[98] Lv H., Zhang S., Wang B., Cui S., Yan J. (2006). Toxicity of cationic lipids and cationic polymers in gene delivery. J. Control. Release.

[99] Hamilton S.E., Simmons C.G., Kathiriya I.S., Corey D.R. (1999). Cellular delivery of peptide nucleic acids and inhibition of human telomerase. Chem. Biol..

[100] Bae Y.M., Kim M.H., Yu G.S., Um B.H., Park H.K., Lee H.-I., Lee K.T., Suh Y.D., Choi J.S. (2014). Enhanced splicing correction effect by an oligo-aspartic acid–PNA conjugate and cationic carrier complexes. J. Control. Release.

[101] Lee J., Ahn H.J. (2018). PEGylated DC-Chol/DOPE cationic liposomes containing KSP siRNA as a systemic siRNA delivery Carrier for ovarian cancer therapy. Biochem. Biophys. Res. Commun..

[102] Hsu S.-H., Yu B., Wang X., Lu Y., Schmidt C.R., Lee R.J., Lee L.J., Jacob S.T., Ghoshal K. (2013). Cationic lipid nanoparticles for therapeutic delivery of siRNA and miRNA to murine liver tumor. Nanomed. Nanotechnol. Biol. Med..

[103] Bulbake U., Doppalapudi S., Kommineni N., Khan W. (2017). Liposomal Formulations in Clinical Use: An Updated Review. Pharmaceutics.

[104] Avitabile C., Accardo A., Ringhieri P., Morelli G., Saviano M., Montagner G., Fabbri E., Gallerani E., Gambari R., Romanelli A. (2015). Incorporation of Naked Peptide Nucleic Acids into Liposomes Leads to Fast and Efficient Delivery. Bioconjugate Chem..

[105] Ringhieri P., Avitabile C., Saviano M., Morelli G., Romanelli A., Accardo A. (2016). The influence of liposomal formulation on the incorporation and retention of PNA oligomers. Colloids Surfaces B Biointerfaces.

[106] Fisher R.K., Mattern-Schain S.I., Best M.D., Kirkpatrick S.S., Freeman M.B., Grandas O.H., Mountain D.J. (2017). Improving the efficacy of liposome-mediated vascular gene therapy via lipid surface modifications. J. Surg. Res..

[107] Ghavami M., Shiraishi T., Nielsen P.E. (2020). Enzyme-Triggered Release of the Antisense Octaarginine-PNA Conjugate from Phospholipase A2 Sensitive Liposomes. ACS Appl. Bio Mater..

[108] Grimaldi N., Andrade F., Segovia N., Tasies L.P.F., Sala S., Veciana J., Ventosa N. (2016). Lipid-based nanovesicles for nanomedicine. Chem. Soc. Rev..

[109] Grijalvo S., Puras G., Zarate J., Sainz-Ramos M., Al Qtaish N., Lopez-Mendez T.B., Mashal M., Attia N., Díaz D.D., Pons R. (2019). Cationic Niosomes as Non-Viral Vehicles for Nucleic Acids: Challenges and Opportunities in Gene Delivery. Pharmaceutics.

[110] Rad A.T., Malik S., Yang L., Oberoi-Khanuja T.K., Nieh M., Bahal R. (2019). A universal discoidal nanoplatform for the intracellular delivery of PNAs. Nanoscale.

[111] Danhier F., Ansorena E., Silva J.M., Coco R., Le Breton A., Préat V. (2012). PLGA-based nanoparticles: An overview of biomedical applications. J. Control. Release.

[112] Makadia H.K., Siegel S.J. (2011). Poly Lactic-co-Glycolic Acid (PLGA) as Biodegradable Controlled Drug Delivery Carrier. Polymers.

[113] Fields R.J., Quijano E., McNeer N.A., Caputo C., Bahal R., Anandalingam K., Egan M.E., Glazer P.M., Saltzman W.M. (2014). Modified Poly (lactic-co-glycolic Acid) Nanoparticles for Enhanced Cellular Uptake and Gene Editing in the Lung. Adv. Heal. Mater..

[114] Park J., Fong P.M., Lu J., Russell K.S., Booth C.J., Saltzman W.M., Fahmy T.M. (2009). PEGylated PLGA nanoparticles for the improved delivery of doxorubicin. Nanomed. Nanotechnol. Biol. Med..

[115] Cheng C.J., Saltzman W.M. (2011). Enhanced siRNA delivery into cells by exploiting the synergy between targeting ligands and cell-penetrating peptides. Biomaterials.

[116] Lynn D.M., Langer R. (2000). Degradable Poly (β-amino esters): Synthesis, Characterization, and Self-Assembly with Plasmid DNA. J. Am. Chem. Soc..

[117] Lynn D.M., Amiji M.M., Langer R. (2001). pH-responsive polymer microspheres: Rapid release of encapsulated material within the range of intracellular pH. Angew. Chem. Int. Ed..

[118] Little S.R., Lynn D.M., Ge Q., Anderson D.G., Puram S.V., Chen J., Eisen H.N., Langer R.S. (2004). From The Cover: Poly-amino ester-containing microparticles enhance the activity of nonviral genetic vaccines. Proc. Natl. Acad. Sci. USA.

[119] Van Vlerken L.E., Duan Z., Little S.R., Seiden M.V., Amiji M. (2008). Biodistribution and Pharmacokinetic Analysis of Paclitaxel and Ceramide Administered in Multifunctional Polymer-Blend Nanoparticles in Drug Resistant Breast Cancer Model. Mol. Pharm..

[120] Fields R.J., Cheng C.J., Quijano E., Weller C., Kristofik N., Duong N., Hoimes C., Egan M.E., Saltzman W.M. (2012). Surface modified poly (β amino ester)-containing nanoparticles for plasmid DNA delivery. J. Control Release.

[121] Cheng C.J., Saltzman W.M. (2012). Polymer Nanoparticle-Mediated Delivery of MicroRNA Inhibition and Alternative Splicing. Mol. Pharm..

[122] Babar I.A., Cheng C.J., Booth C.J., Liang X., Weidhaas J.B., Saltzman W.M., Slack F.J. (2012). Nanoparticle-based therapy in an in vivo microRNA-155 (miR-155)-dependent mouse model of lymphoma. Proc. Natl. Acad. Sci. USA.

[123] Peer D., Karp J.M., Hong S., Farokhzad O.C., Margalit R., Langer R. (2007). Nanocarriers as an emerging platform for cancer therapy. Nat. Nanotechnol..

[124] Fontana L., Fiori M.E., Albini S., Cifaldi L., Giovinazzi S., Forloni M., Boldrini R., Donfrancesco A., Federici V., Giacomini P. (2008). Antagomir-17-5p Abolishes the Growth of Therapy-Resistant Neuroblastoma through p21 and BIM. PLoS ONE.

[125] Ma L., Reinhardt F., Pan E., Soutschek J., Bhat B., Marcusson E.G., Teruya-Feldstein J., Bell G.W., Weinberg R.A. (2010). Therapeutic silencing of miR-10b inhibits metastasis in a mouse mammary tumor model. Nat. Biotechnol..

[126] Gupta A., Quijano E., Liu Y., Bahal R., Scanlon S.E., Song E., Hsieh W.-C., Braddock D.E., Ly D.H., Saltzman W.M. (2017). Anti-tumor Activity of miniPEG-γ-Modified PNAs to Inhibit MicroRNA-210 for Cancer Therapy. Mol. Ther. Nucleic Acids.

[127] Grosso S., Doyen J., Parks S.K., Bertero T., Paye A., Cardinaud B., Gounon P., Lacas-Gervais S., Noël A., Pouysségur J. (2013). MiR-210 promotes a hypoxic phenotype and increases radioresistance in human lung cancer cell lines. Cell Death Dis..

[128] Ho A.S., Huang X., Cao H., Christman-Skieller C., Bennewith K., Le Q.-T., Koong A.C. (2010). Circulating miR-210 as a Novel Hypoxia Marker in Pancreatic Cancer. Transl. Oncol..

[129] Qin Q., Furong W., Li B. (2014). Multiple functions of hypoxia-regulated miR-210 in cancer. J. Exp. Clin. Cancer Res..

[130] Sahu B., Sacui I., Rapireddy S., Zanotti K.J., Bahal R., Armitage A.A., Ly D.H. (2011). Synthesis and characterization of conformationally peptide nucleic acids with superior hybridization properties and water solubility. J. Org. Chem..

[131] Dragulescu-Andrasi A., Rapireddy S., Frezza B.M., Gayathri C., Gil R.R., Ly D.H. (2006). A Simple γ-Backbone Modification Preorganizes Peptide Nucleic Acid into a Helical Structure. J. Am. Chem. Soc..

[132] Bahal R., McNeer N.A., Ly D.H., Saltzman W.M., Glazer P.M. (2013). Nanoparticle for delivery of antisense γPNA oligomers targeting CCR5. Artif. DNA PNA XNA.

[133] Samson M., Libert F., Doranz B.J., Rucker J., Liesnard C., Farber C.-M., Saragosti S., Lapouméroulie C., Cognaux J., Forceille C. (1996). Resistance to HIV-1 infection in Caucasian individuals bearing mutant alleles of the CCR-5 chemokine receptor gene. Nat. Cell Biol..

[134] Ricciardi A.S., Quijano E., Putman R., Saltzman W.M., Glazer P.M. (2018). Peptide Nucleic Acids as a Tool for Site-Specific Gene Editing. Molecules.

[135] Schleifman E.B., Bindra R., Leif J., Del Campo J., Rogers F.A., Uchil P.D., Kutsch O., Shultz L.D., Kumar P., Greiner D.L. (2011). Targeted Disruption of the CCR5 Gene in Human Hematopoietic Stem Cells Stimulated by Peptide Nucleic Acids. Chem. Biol..

[136] Kaihatsu K., Shah R.H., Zhao X., Corey D.R. (2003). Extending Recognition by Peptide Nucleic Acids (PNAs): Binding to Duplex DNA and Inhibition of Transcription by Tail-Clamp PNA−Peptide Conjugates†. Biochemicals.

[137] Bentin T., Larsen H.J., Nielsen P.E. (2003). Combined Triplex/Duplex Invasion of Double-Stranded DNA by “Tail-Clamp” Peptide Nucleic Acid†. Biochemicals.

[138] Bahal R., Sahu B., Rapireddy S., Lee C.-M., Ly D.H. (2011). Sequence-Unrestricted, Watson-Crick Recognition of Double Helical B-DNA by (R)-MiniPEG-γPNAs. ChemBioChem.

[139] Yeh J.I., Shivachev B., Rapireddy S., Crawford M.J., Gil R.R., Du S., Madrid M., Ly D.H. (2010). Crystal Structure of Chiral γPNA with Complementary DNA Strand: Insights into the Stability and Specificity of Recognition and Conformational Preorganization. J. Am. Chem. Soc..

[140] Bahal R., Quijano E., McNeer N.A., Liu Y., Bhunia D.C., Lopez-Giraldez F., Fields R.J., Saltzman W.M., Ly D.H., Glazer P.M. (2014). Single-Stranded γPNAs for In Vivo Site-Specific Genome Editing via Watson-Crick Recognition. Curr. Gene Ther..

[141] Chin J.Y., Kuan J.Y., Lonkar P.S., Krause D.S., Seidman M.M., Peterson K.R., Nielsen P.E., Kole R., Glazer P.M. (2008). Correction of a splice-site mutation in the beta-globin gene stimulated by triplex-forming peptide nucleic acids. Proc. Natl. Acad. Sci. USA.

[142] A McNeer N., Chin J.Y., Schleifman E.B., Fields R.J., Glazer P.M., Saltzman W.M. (2011). Nanoparticles Deliver Triplex-forming PNAs for Site-specific Genomic Recombination in CD34+ Human Hematopoietic Progenitors. Mol. Ther..

[143] McNeer N.A., Schleifman E.B., Cuthbert A., A Brehm M., Jackson A., Cheng C., Anandalingam K., Kumar P., Shultz L.D., Greiner D.L. (2012). Systemic delivery of triplex-forming PNA and donor DNA by nanoparticles mediates site-specific genome editing of human hematopoietic cells in vivo. Gene Ther..

[144] Ricciardi A.S., Bahal R., Farrelly J.S., Quijano E., Bianchi A.H., Luks V.L., Putman R., López-Giráldez F., Coşkun S., Song E. (2018). In utero nanoparticle delivery for site-specific genome editing. Nat. Commun..

[145] Zhang X.-H., Tee L.Y., Wang X.-G., Huang Q.-S., Yang S.-H. (2015). Off-target effects in CRISPR/Cas9-mediated genome engineering. Mol. Ther. Nucleic Acids.

[146] Liu R., A Paxton W., Choe S., Ceradini D., Martin S.R., Horuk R., E MacDonald M., Stuhlmann H., A Koup R., Landau N.R. (1996). Homozygous Defect in HIV-1 Coreceptor Accounts for Resistance of Some Multiply-Exposed Individuals to HIV-1 Infection. Cell.

[147] Schleifman E.B., McNeer N.A., Jackson A., Yamtich J., A Brehm M., Shultz L.D., Greiner D.L., Kumar P., Saltzman W.M., Glazer P.M. (2013). Site-specific Genome Editing in PBMCs With PLGA Nanoparticle-delivered PNAs Confers HIV-1 Resistance in Humanized Mice. Mol. Ther. Nucleic Acids.

[148] E Perez E., Wang J., Miller J.C., Jouvenot Y., A Kim K., Liu O., Wang N., Lee G., Bartsevich V.V., Lee Y.-L. (2008). Establishment of HIV-1 resistance in CD4+ T cells by genome editing using zinc-finger nucleases. Nat. Biotechnol..

[149] Rowe S.M., Miller S., Sorscher E.J. (2005). Cystic Fibrosis. N. Engl. J. Med..

[150] McNeer N.A., Anandalingam K., Fields R.J., Caputo C., Kopic S., Gupta A., Quijano E., Polikoff L., Kong Y., Bahal R. (2015). Nanoparticles that deliver triplex-forming peptide nucleic acid molecules correct F508del CFTR in airway epithelium. Nat. Commun..

[151] Van Nostrum C.F. (2011). Covalently cross-linked amphiphilic block copolymer micelles. Soft Matter.

[152] Elsabahy M., Wooley K.L. (2012). Design of polymeric nanoparticles for biomedical delivery applications. Chem. Soc. Rev..

[153] Fang H., Zhang K., Shen G., Wooley K.L., Taylor J.-S.A. (2009). Cationic Shell-Cross-Linked Knedel-like (cSCK) Nanoparticles for Highly Efficient PNA Delivery. Mol. Pharm..

[154] Turner J.L., Becker M.L., Li X., Taylor J.-S.A., Wooley K.L. (2005). PNA-directed solution- and surface-assembly of shell crosslinked (SCK) nanoparticle conjugates. Soft Matter.

[155] Zhang K., Fang H., Wang Z., Taylor J.-S.A., Wooley K.L. (2009). Cationic shell-crosslinked knedel-like nanoparticles for highly efficient gene and oligonucleotide transfection of mammalian cells. Biomaterials.

[156] Kang S.-H., Cho M.-J., Kole R. (1998). Up-Regulation of Luciferase Gene Expression with Antisense Oligonucleotides: Implications and Applications in Functional Assay Development†. Biochemicals.

[157] Boussif O., Lezoualc’H F., Zanta M.A., Mergny M.D., Scherman D., Demeneix B., Behr J.P. (1995). A versatile vector for gene and oligonucleotide transfer into cells in culture and in vivo: Polyethylenimine. Proc. Natl. Acad. Sci. USA.

[158] Johnson E.R., Matthay M.A. (2010). Acute Lung Injury: Epidemiology, Pathogenesis, and Treatment. J. Aerosol Med. Pulm. Drug Deliv..

[159] Hosogi S., Iwasaki Y., Yamada T., Komatani-Tamiya N., Hiramatsu A., Kohno Y., Ueda M., Arimoto T., Marunaka Y. (2008). Effect of inducible nitric oxide synthase on apoptosis in Candida-induced acute lung injury. Biomed. Res..

[160] Mehta S. (2005). The effects of nitric oxide in acute lung injury. Vasc. Pharmacol..

[161] Wang Z., Zhang K., Wooley K.L., Taylor J.-S.A. (2012). Imaging mRNA Expression in Live Cells via PNA·DNA Strand Displacement-Activated Probes. J. Nucleic Acids.

[162] Kuhn H., Demidov V.V., Gildea B.D., Fiandaca M.J., Coull J.C., Frank-Kamenetskii M.D. (2001). PNA Beacons for Duplex DNA. Antisense Nucleic Acid Drug Dev..

[163] Tyagi S. (2009). Imaging intracellular RNA distribution and dynamics in living cells. Nat. Methods.

[164] Shrestha R., Shen Y., Pollack K.A., Taylor J.-S.A., Wooley K.L. (2012). Dual Peptide Nucleic Acid- and Peptide-Functionalized Shell Cross-Linked Nanoparticles Designed to Target mRNA toward the Diagnosis and Treatment of Acute Lung Injury. Bioconjugate Chem..

[165] Shen Y., Shrestha R., Ibricevic A., Gunsten S.P., Welch M.J., Wooley K.L., Brody S.L., Taylor J.-S.A., Liu Y. (2013). Antisense peptide nucleic acid-functionalized cationic nanocomplex for in vivo mRNA detection. Interface Focus.

[166] Rinaudo M. (2006). Chitin and chitosan: Properties and applications. Prog. Polym. Sci..

[167] Aranaz I., Harris R., Heras A. (2010). Chitosan Amphiphilic Derivatives. Chemistry and Applications. Curr. Org. Chem..

[168] Liu C., Wang J., Huang S., Yu L., Wang Y., Chen H., Wang D. (2018). Self-assembled nanoparticles for cellular delivery of peptide nucleic acid using amphiphilic *N,N,N*-trimethyl-O-alkyl chitosan derivatives. J. Mater. Sci. Mater. Electron..

[169] Lülf H., Bertucci A., Septiadi D., Corradini R., De Cola L. (2014). Multifunctional Inorganic Nanocontainers for DNA and Drug Delivery into Living Cells. Chem. A Eur. J..

[170] Bertucci A., Lülf H., Septiadi D., Manicardi A., Corradini R., De Cola L. (2014). Intracellular Delivery of Peptide Nucleic Acid and Organic Molecules Using Zeolite-L Nanocrystals. Adv. Heal. Mater..

[171] Bertucci A. (2015). Alessandro Bertucci Hybrid Organic-Inorganic Interfaces for Biomedical Applications. Ph.D. Thesis.

[172] Vallet-Regi M., Rámila A., Del Real R.P., Pérez-Pariente J. (2001). A New Property of MCM-41: Drug Delivery System. Chem. Mater..

[173] Climent E., Martínez-Máñez R., Sancenón F., Marcos M.D., Soto J., Maquieira Á., Amorós P. (2010). Controlled Delivery Using Oligonucleotide-Capped Mesoporous Silica Nanoparticles. Angew. Chem. Int. Ed..

[174] Mackowiak S.A., Schmidt A., Weiss V., Argyo C., Von Schirnding C., Bein T., Bräuchle C. (2013). Targeted Drug Delivery in Cancer Cells with Red-Light Photoactivated Mesoporous Silica Nanoparticles. Nano Lett..

[175] Cauda V.A., Argyo C., Bein T. (2010). Impact of different PEGylation patterns on the long-term bio-stability of colloidal mesoporous silica nanoparticles. J. Mater. Chem..

[176] Sauer A.M., Schlossbauer A., Ruthardt N., Cauda V.A., Bein T., Braäuchle C. (2010). Role of Endosomal Escape for Disulfide-Based Drug Delivery from Colloidal Mesoporous Silica Evaluated by Live-Cell Imaging. Nano Lett..

[177] Zhang Q., Wang X., Li P.-Z., Nguyen K.T., Wang X., Luo Z., Zhang H., Tan N.S., Zhao Y. (2013). Biocompatible, Uniform, and Redispersible Mesoporous Silica Nanoparticles for Cancer-Targeted Drug Delivery In Vivo. Adv. Funct. Mater..

[178] Kruk M., Cao L. (2007). Pore Size Tailoring in Large-Pore SBA-15 Silica Synthesized in the Presence of Hexane. Langmuir.

[179] Moeller K., Müller K., Engelke H., Bräuchle C., Wagner E., Bein T. (2016). Highly efficient siRNA delivery from core–shell mesoporous silica nanoparticles with multifunctional polymer caps. Nanoscale.

[180] Chang J.-H., Tsai P.-H., Chen W., Chiou S.-H., Mou C.-Y. (2017). Dual delivery of siRNA and plasmid DNA using mesoporous silica nanoparticles to differentiate induced pluripotent stem cells into dopaminergic neurons. J. Mater. Chem. B.

[181] Bertucci A., Prasetyanto E.A., Septiadi D., Manicardi A., Brognara E., Gambari R., Corradini R., De Cola L. (2015). Combined Delivery of Temozolomide and Anti-miR221 PNA Using Mesoporous Silica Nanoparticles Induces Apoptosis in Resistant Glioma Cells. Small.

[182] Ma X., Devi G., Qu Q., Toh D.-F.K., Chen G., Zhao Y. (2014). Intracellular Delivery of Antisense Peptide Nucleic Acid by Fluorescent Mesoporous Silica Nanoparticles. Bioconjugate Chem..

[183] Prasetyanto E.A., Bertucci A., Septiadi D., Corradini R., Castro-Hartmann P., De Cola L. (2016). Breakable Hybrid Organosilica Nanocapsules for Protein Delivery. Angew. Chem. Int. Ed..

[184] Maggini L., Cabrera I., Ruiz-Carretero A., Prasetyanto E.A., Robinet E., De Cola L. (2016). Breakable mesoporous silica nanoparticles for targeted drug delivery. Nanoscale.

[185] Canham L.T. (1990). Silicon quantum wire array fabricaiton by electrochemical. Appl. Phys. Lett..

[186] Canham L.T. (1995). Bioactive Silicon Structure Fabrication through Nanoetching Techniques. Adv. Mater..

[187] Bertucci A., Kim K.-H., Kang J., Zuidema J.M., Lee S.H., Kwon E.J., Kim D., Howell S.B., Ricci F., Ruoslahti E. (2019). Tumor-Targeting, MicroRNA-Silencing Porous Silicon Nanoparticles for Ovarian Cancer Therapy. ACS Appl. Mater. Interfaces.

[188] Beavers K.R., Mares J.W., Swartz C.M., Zhao Y., Weiss S.M., Duvall C.L. (2014). In Situ Synthesis of Peptide Nucleic Acids in Porous Silicon for Drug Delivery and Biosensing. Bioconjugate Chem..

[189] Beavers K.R., Werfel T.A., Shen T., Kavanaugh T.E., Kilchrist K.V., Mares J.W., Fain J.S., Wiese C.B., Vickers K.C., Weiss S.M. (2016). Porous Silicon and Polymer Nanocomposites for Delivery of Peptide Nucleic Acids as Anti-MicroRNA Therapies. Adv. Mater..

[190] Park J.-H., Gu L., Von Maltzahn G., Ruoslahti E., Bhatia S.N., Sailor M.J. (2009). Biodegradable luminescent porous silicon nanoparticles for in vivo applications. Nat. Mater..

[191] Kang J., Joo J., Kwon E.J., Skalak M., Hussain S., She Z.-G., Ruoslahti E., Bhatia S.N., Sailor M.J. (2016). Self-Sealing Porous Silicon-Calcium Silicate Core-Shell Nanoparticles for Targeted siRNA Delivery to the Injured Brain. Adv. Mater..

[192] Kwon E.J., Skalak M., Bertucci A., Braun G., Ricci F., Ruoslahti E., Sailor M.J., Bhatia S.N. (2017). Porous Silicon Nanoparticle Delivery of Tandem Peptide Anti-Infectives for the Treatment ofPseudomonas aeruginosaLung Infections. Adv. Mater..

[193] Kelly I.B., Fletcher R.B., McBride J.R., Weiss S.M., Duvall C.L. (2020). Tuning Composition of Polymer and Porous Silicon Composite Nanoparticles for Early Endosome Escape of Anti-microRNA Peptide Nucleic Acids. ACS Appl. Mater. Interfaces.

[194] Evans B.C., Fletcher B., Kilchrist K.V., Dailing E.A., Mukalel A.J., Colazo J.M., Oliver M., Cheung-Flynn J., Brophy C.M., Tierney J.W. (2019). An anionic, endosome-escaping polymer to potentiate intracellular delivery of cationic peptides, biomacromolecules, and nanoparticles. Nat. Commun..

[195] Yu Z., Hu P., Xu Y., Bao Q., Ni D., Wei C., Shi J. (2020). Efficient Gene Therapy of Pancreatic Cancer via a Peptide Nucleic Acid (PNA)-Loaded Layered Double Hydroxides (LDH) Nanoplatform. Small.

[196] Galli M., Guerrini A., Cauteruccio S., Thakare P., Dova D., Orsini F., Arosio P., Carrara C., Sangregorio C., Lascialfari A. (2017). Superparamagnetic iron oxide nanoparticles functionalized by peptide nucleic acids. RSC Adv..

[197] Prencipe G., Maiorana S., Verderio P., Colombo M., Fermo P., Caneva E., Prosperi D., Licandro E. (2009). Magnetic peptide nucleic acids for DNA targeting. Chem. Commun..

[198] Ghaffari E., Rezatofighi S.E., Ardakani M.R., Rastegarzadeh S. (2019). Delivery of antisense peptide nucleic acid by gold nanoparticles for the inhibition of virus replication. Nanomedicine.

[199] Lee J., Kim J., Kim S., Min D.-H. (2016). Biosensors based on graphene oxide and its biomedical application. Adv. Drug Deliv. Rev..

[200] Kim J., Park S.-J., Min D.-H. (2016). Emerging Approaches for Graphene Oxide Biosensor. Anal. Chem..

[201] Oh H.J., Kim J., Park H., Chung S., Hwang D.W., Lee D.S. (2019). Graphene-oxide quenching-based molecular beacon imaging of exosome-mediated transfer of neurogenic miR-193a on microfluidic platform. Biosens. Bioelectron..

[202] Ryoo S.-R., Lee J., Yeo J., Na H.-K., Kim Y.-K., Jang H., Lee J.H., Han S.W., Lee Y., Kim V.N. (2013). Quantitative and Multiplexed MicroRNA Sensing in Living Cells Based on Peptide Nucleic Acid and Nano Graphene Oxide (PANGO). ACS Nano.

[203] Hwang D.W., Choi Y.R., Kim H., Park H.Y., Kim K.W., Kim M.Y., Park C.-K., Lee D. (2019). Graphene oxide-quenching-based fluorescence in situ hybridization (G-FISH) to detect RNA in tissue: Simple and fast tissue RNA diagnostics. Nanomed. Nanotechnol. Biol. Med..

[204] Lee J.-S., Kim S., Na H.-K., Min D.-H. (2016). MicroRNA-Responsive Drug Release System for Selective Fluorescence Imaging and Photodynamic Therapy In Vivo. Adv. Heal. Mater..

[205] Baek A., Baek Y.M., Kim H.-M., Jun B.-H., Kim D.-E. (2018). Polyethylene Glycol-Engrafted Graphene Oxide as Biocompatible Materials for Peptide Nucleic Acid Delivery into Cells. Bioconjugate Chem..

[206] Hwang D.W., Kim H.Y., Li F., Park J.Y., Kim D., Park J.H., Han H.S., Byun J.W., Lee Y.-S., Jeong J.M. (2017). In vivo visualization of endogenous miR-21 using hyaluronic acid-coated graphene oxide for targeted cancer therapy. Biomaterials.

[207] Liao X., Wang Q., Ju H. (2015). A peptide nucleic acid-functionalized carbon nitride nanosheet as a probe for in situ monitoring of intracellular microRNA. Analyst.

[208] Gaillard C., Girard H.A., Falck C., Paget V., Simic V., Ugolin N., Bergonzo P., Chevillard S., Arnault J.C. (2014). Peptide nucleic acid–nanodiamonds: Covalent and stable conjugates for DNA targeting. RSC Adv..

[209] Arayachukiat S., Seemork J., Pan-In P., Amornwachirabodee K., Sangphech N., Sansureerungsikul T., Sathornsantikun K., Vilaivan C., Shigyou K., Pienpinijtham P. (2015). Bringing Macromolecules into Cells and Evading Endosomes by Oxidized Carbon Nanoparticles. Nano Lett..

[210] Tarvirdipour S., Huang X., Mihali V., Schoenenberger C.-A., Palivan C.G. (2020). Peptide-Based Nanoassemblies in Gene Therapy and Diagnosis: Paving the Way for Clinical Application. Molecules.

[211] Macadangdang B., Zhang N., Lund P.E., Marple A.H., Okabe M., Gottesman M.M., Appella D.H., Kimchi-Sarfaty C. (2011). Inhibition of Multidrug Resistance by SV40 Pseudovirion Delivery of an Antigene Peptide Nucleic Acid (PNA) in Cultured Cells. PLoS ONE.

[212] Morris M.C., Depollier J., Mery J., Heitz F., Divita G. (2001). A peptide carrier for the delivery of biologically active proteins into mammalian cells. Nat. Biotechnol..

[213] Morris M.C., Chaloin L., Choob M., Archdeacon J., Heitz F., Divita G. (2004). Combination of a new generation of PNAs with a peptide-based carrier enables efficient targeting of cell cycle progression. Gene Ther..

[214] Galli V., Sadhu K.K., Masi D., Saarbach J., Roux A., Winssinger N. (2019). Caprin-1 Promotes Cellular Uptake of Nucleic Acids with Backbone and Sequence Discrimination. Helv. Chim. Acta.

[215] Valero J., Shiraishi T., De Mendoza J., Nielsen P.E. (2015). Cellular Antisense Activity of PNA-Oligo (bicycloguanidinium) Conjugates Forming Self-Assembled Nanoaggregates. ChemBioChem.

[216] Ghavami M., Shiraishi T., Nielsen P.E. (2019). Cooperative Cellular Uptake and Activity of Octaarginine Antisense Peptide Nucleic acid (PNA) Conjugates. Biomolecules.

[217] Sansone F., Dudič M., Donofrio G., Rivetti C., Baldini L., Casnati A., Cellai A.S., Ungaro R. (2006). DNA Condensation and Cell Transfection Properties of Guanidinium Calixarenes: Dependence on Macrocycle Lipophilicity, Size, and Conformation. J. Am. Chem. Soc..

[218] Gasparello J., Lomazzi M., Papi C., D’Aversa E., Sansone F., Casnati A., Donofrio G., Gambari R., Finotti A. (2019). Efficient Delivery of MicroRNA and AntimiRNA Molecules Using an Argininocalix[4]arene Macrocycle. Mol. Ther. Nucleic Acids.

[219] Gasparello J., Manicardi A., Casnati A., Corradini R., Gambari R., Finotti A., Sansone F. (2019). Efficient cell penetration and delivery of peptide nucleic acids by an argininocalix[4]arene. Sci. Rep..

[220] Tomassi S., Ieranò C., Mercurio M.E., Nigro E., Daniele A., Russo R., Chambery A., Baglivo I., Pedone P.V., Rea G. (2018). Cationic nucleopeptides as novel non-covalent carriers for the delivery of peptide nucleic acid (PNA) and RNA oligomers. Bioorganic Med. Chem..

[221] Seeman N.C., Sleiman H.F. (2018). DNA nanotechnology. Nat. Rev. Mater..

[222] Hong F., Zhang F., Liu Y., Yan H. (2017). DNA Origami: Scaffolds for Creating Higher Order Structures. Chem. Rev..

[223] Zhang D.Y., Seelig G. (2011). Dynamic DNA nanotechnology using strand-displacement reactions. Nat. Chem..

[224] Hu Q., Li H., Wang L., Gu H.-Z., Fan C. (2018). DNA Nanotechnology-Enabled Drug Delivery Systems. Chem. Rev..

[225] Zhao X.-L., Chen B.-C., Han J.-C., Wei L., Pan X.-B. (2015). Delivery of cell-penetrating peptide-peptide nucleic acid conjugates by assembly on an oligonucleotide scaffold. Sci. Rep..

[226] Lo S.L., Wang S. (2008). An endosomolytic Tat peptide produced by incorporation of histidine and cysteine residues as a nonviral vector for DNA transfection. Biomaterials.

[227] Readman J.B., Dickson G., Coldham N.G. (2017). Tetrahedral DNA Nanoparticle Vector for Intracellular Delivery of Targeted Peptide Nucleic Acid Antisense Agents to Restore Antibiotic Sensitivity in Cefotaxime-ResistantEscherichia coli. Nucleic Acid Ther..

[228] Readman J.B., Dickson G., Coldham N.G. (2016). Translational Inhibition of CTX-M Extended Spectrum β-Lactamase in Clinical Strains of Escherichia coli by Synthetic Antisense Oligonucleotides Partially Restores Sensitivity to Cefotaxime. Front. Microbiol..

[229] Zhang Y., Ma W., Zhu Y., Shi S., Li Q.-S., Mao C., Zhao D., Shi J., Li W., Wang L. (2018). Inhibiting Methicillin-Resistant Staphylococcus aureus by Tetrahedral DNA Nanostructure-Enabled Antisense Peptide Nucleic Acid Delivery. Nano Lett..

[230] Haydon D.J., Stokes N.R., Ure R., Galbraith G., Bennett J.M., Brown D.R., Baker P.J., Barynin V.V., Rice D.W., Sedelnikova S.E. (2008). An Inhibitor of FtsZ with Potent and Selective Anti-Staphylococcal Activity. Science.

[231] Mercurio M.E., Tomassi S., Gaglione M., Russo R., Chambery A., Lama S., Stiuso P., Cosconati S., Novellino E., Di Maro S. (2016). Switchable Protecting Strategy for Solid Phase Synthesis of DNA and RNA Interacting Nucleopeptides. J. Org. Chem..

